# Understanding structural variability in proteins using protein structural networks

**DOI:** 10.1016/j.crstbi.2022.04.002

**Published:** 2022-04-27

**Authors:** Vasam Manjveekar Prabantu, Vasundhara Gadiyaram, Saraswathi Vishveshwara, Narayanaswamy Srinivasan

**Affiliations:** a Molecular Biophysics Unit, Indian Institute of Science, Bangalore, India; b National Centre for Biological Sciences, TIFR, Bangalore, India

**Keywords:** Structural variability, Protein structural networks, Protein structure comparison, Protein classification, Edge-weight variance

## Abstract

Proteins perform their function by accessing a suitable conformer from the ensemble of available conformations. The conformational diversity of a chosen protein structure can be obtained by experimental methods under different conditions. A key issue is the accurate comparison of different conformations. A gold standard used for such a comparison is the root mean square deviation (RMSD) between the two structures. While extensive refinements of RMSD evaluation at the backbone level are available, a comprehensive framework including the side chain interaction is not well understood. Here we employ protein structure network (PSN) formalism, with the non-covalent interactions of side chain, explicitly treated. The PSNs thus constructed are compared through graph spectral method, which provides a comparison at the local and at the global structural level. In this work, PSNs of multiple crystal conformers of single-chain, single-domain proteins, are subject to pair-wise analysis to examine the dissimilarity in their network topologies and in order to determine the conformational diversity of their native structures. This information is utilized to classify the structural domains of proteins into different categories. It is observed that proteins typically tend to retain structure and interactions at the backbone level. However, some of them also depict variability in either their overall structure or only in their inter-residue connectivity at the sidechain level, or both. Variability of sub-networks based on solvent accessibility and secondary structure is studied. The types of specific interactions are found to contribute differently to structure variability. An ensemble analysis by computing the mathematical variance of edge-weights across multiple conformers provided information on the contribution to overall variability from each edge of the PSN. Interactions that are highly variable are identified and their impact on structure variability has been discussed with the help of a case study. The classification based on the present side-chain network-based studies provides a framework to correlate the structure-function relationships in protein structures.

## Highlights

• Monomeric, single domain protein structures can exhibit non-rigid behaviour and be highly variable.
• The comparison of protein structural networks can better discriminate conformations with similar backbones.
• Specific interactions between solvent accessible and inaccessible residues are poorly preserved.
• Network edge-variation offers insights on which interacting residues are likely to influence their dynamics and function.
• These side-chain network-based studies provide a framework to correlate protein structure-function relationships.

## Graphical abstract

**Figure:**
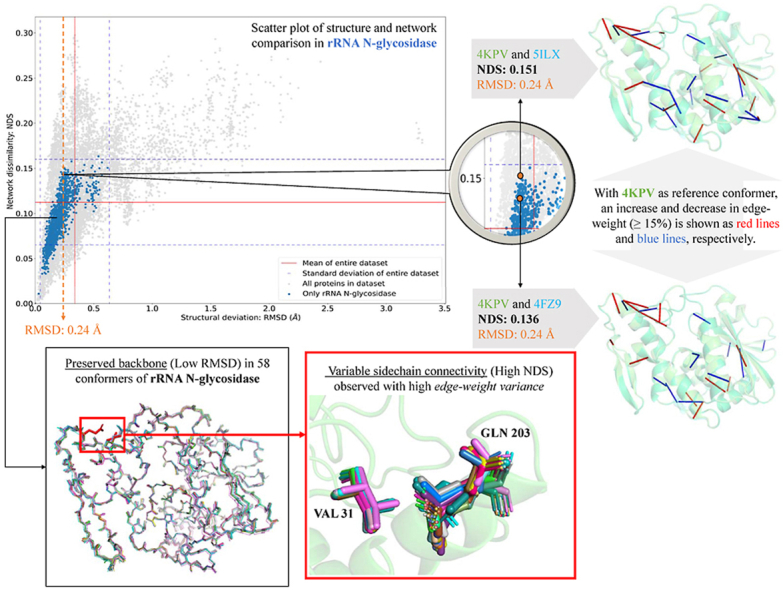

## 1 Introduction

The newly synthesized protein sequences in the cell adopt unique three-dimensional structures (Anfinsen, 1973) to perform their functions. The native structures thus obtained are stabilized by various non-covalent interactions like van der Waals, electrostatic interactions and hydrogen bonds. However, flexibility in its structure allows for it to perform function (Frauenfelder et al., 2007). For instance, a complex function such as open-close motions for transporting ligands across cell membranes or a simple function such as binding of a ligand, require the rearrangement of atomic interactions within the protein structure.

Structures of proteins have been determined using methods like X-ray crystallography, Cryo-EM and NMR in different functional forms. In the case of crystal structures, they are also determined in different crystalline states, different crystallization conditions, etc. Three dimensional coordinates, thus obtained, represent snapshots in various conditions. Even though some of the native conformations of a protein are not crystallisable or have not yet been crystallised, differences observed in the available tertiary structures under various conditions reflect intrinsic flexibility in its overall structure. Depending on the inherent dynamics of the protein, variations in the 3D structure of a protein may be as small as subtle sidechain variation or a very large deviation of the backbone conformation.

The pioneering work of GN Ramachandran (Ramachandran et al., 1963) on the (φ-ψ) map that describes the backbone conformation, has played a key role in our understanding of the protein structure. A refined structure can be generated by providing information on sidechain conformations. With an increase in high resolution protein structural data, rotamer libraries of sidechain conformations have become available for modelling protein structures (Dunbrack, 2002). A recent study of sidechain conformational preferences in monopeptides (Rose, 2019), provides insights akin to Ramachandran (φ-ψ) map of secondary structures. Thus, the allowed and the preferred conformations of the backbone polypeptide chain and that of the connected sidechains are well understood. Another crucial factor required to understand the global topology of protein structures, is the interaction between neighbouring sidechains. The present study focuses on the interactions between spatially proximal sidechains, which provide a global sidechain connectivity map in protein structures.

The alteration of protein structure due to dynamics is characterized by variation of inter-residue interactions. The information of inter-residue interactions is vital to understanding protein function and is used in studying protein folding and stability (Gromiha and Selvaraj, 2004; Baker, 2000), homology detection (Bhattacharya et al., 2021), prediction of protein structures (Yang et al., 2020), and several other aspects. From a topological perspective, intra-protein interactions between spatially proximal residues can be represented on a graph using edges where the residues are represented as nodes. The node-edge representation of a protein structure is commonly known as a protein structural network (PSN). PSNs are used to analyse structural organisation in proteins based on topological distance, nature of interactions, solvent accessibility, geometry, charge, energy and many other features (Vijayabaskar and Vishveshwara, 2010; Bhattacharyya et al., 2015). It allows for the survey of non-covalent interactions like hydrogen bonds, ionic, hydrophobic, and van der Waals interactions (Vishveshwara et al., 2002). There are several advantages of using PSNs to gather structure and functional information such as analysing subtle conformational changes due to ligand binding or for identifying communication paths of allosteric effects (Costanzi, 2016; Guarnera et al., 2017; Brinda and Vishveshwara, 2005; Amitai et al., 2004). Aside from intra-protein interactions they can also be used in the investigation of protein-ligand and protein-protein interactions (Taylor, 2013).

Several tools have been developed to compare and quantify the difference between PSNs (Schieber et al., 2017; Faisal et al., 2017; Malod-Dognin and Pr ž ulj, 2014). The connectivity information in the form of binary matrix is computationally easy to handle even for large number of comparisons. Hence, the strength of interactions has often been digitised as zero or one based on a selection criterion. Physics based approach such as the percolation transition point is a method used for selecting the optimal edge-weight to make the matrix binary. In the comparison of PSNs, graph-spectra based methods are very useful as they depict the global arrangement of nodes and their connectivity with minimal loss of information (Deb et al., 2009). Several methods have employed the use of graph spectra for the comparison of protein structures (Chakrabarty and Parekh, 2014a, 2014b; Bhattacharyya et al., 2013).

Advancement in algorithms and computing power has led to the development of graph spectral methods to handle weighted matrices. This allows us to analyse PSNs using graph spectral features, incorporating the edge differences at the local level and the differences in modes of clustering at the global level. One such approach is the comparison of networks using the network similarity score (NSS) (Gadiyaram et al., 2017), which also serves to quantify the dissimilarity between a pair of PSNs. NSS can provide the alterations in spatial proximity between sequentially non-adjacent residues along with any alterations in the clustering of residues at the global (tertiary structure) as well as at the local level (sidechain interactions), making it robust and sensitive to minute changes between the compared networks. Thus, NSS is sensitive to minute changes in networks, making it a robust method to perform quantitative comparison between near-similar protein structures. The protocol has been earlier employed in the validation of protein structure models (Ghosh et al., 2017) and for protein structure comparison (Gadiyaram et al., 2019). We have extensively made use of this method in the current work for studying dissimilarity between structural networks of proteins and have termed the measure as the network dissimilarity score (NDS).

The main focus of this work is to characterise the extent of diversity in structures of a protein (or its ligand-complex) under varying conditions obtained from multiple crystal conformers, using the network formalism. We analyse inter-residue interactions within each protein by employing PSN that are constructed from coordinates of all non-hydrogen atoms from multiple crystal structures available for a given protein. The deviation in backbone is measured using the conventional root mean square deviation (RMSD) and changes in structural network are studied using the NDS. It is known that the 3D structure of some proteins may have several regions that are rigid while other regions, generally relating to their function, may show mobility (Burra et al., 2009). Likewise, in the analysis of protein structures that are independent of external interactions we observe that the nature of structural variability can range from strongly rigid behaviour to being highly dynamic and undergoing large conformational changes. These structure variations within the protein have been studied, since they are also determinant factors of their function. Based on these studies, we have categorised the protein structures into several groups and have discussed their implications. The methodology is described in the next section and the Results and Discussion are presented in section 3, 4 respectively.

## 2 Materials and methods

### 2.1 Multiple crystal structures of single domain monomeric proteins

The dataset is assembled by collecting all full-length structures of single chain protein structures from the protein data bank (PDB) (Berman et al., 2000) that are obtained using X-ray diffraction. A selection criterion of better than 3 ​Å resolution with Rfree and Rwork better than 30% and 25% respectively is considered. Any chain with more than a single domain (as defined on SCOPe) (Fox et al., 2014)^,^ (Chandonia et al., 2019) is not included. All structures with missing residue information or mutations in the non-terminal regions of the sequence are removed. An adequate number of structures for each protein is necessary to study structural variability. A threshold of five PDB entries for a protein is included for further analysis. The dataset assembled consists of 913 PDB entries of 56 proteins, with the number of crystal conformers for each protein ranging from six to fifty-nine. Supplementary Table 1 lists the details of all proteins in the dataset.

### 2.2 Protein structural network construction

The protein structural network (PSN) of a crystal conformer is constructed from the 3D structure coordinates retrieved from the protein data bank (PDB). The amino acid residues in the structure are considered as nodes and undirected weighted edges are drawn between each pair of interacting residues based on the strength of their interactions. The edge-weight between nodes in the PSN is equivalent to the fraction of the number of contacts made by proximal atoms (between *i*th and *j*th residues of the given protein), with respect to the maximum number of such contacts that are found between the pair of corresponding amino acids from the entire dataset. Such a ratio translates the interaction strength between the two connected residues into edge-weight between the two corresponding nodes. A proximity-based measure of edge-weight ($I_{ij}$) between any two sequentially non-adjacent residues i and j is computed using Equation (1) (Bhattacharyya et al., 2015).

Equation 1 $$I_{ij}=\frac{Number\;of\;proximal\;atoms\;between\;amino\;acids\;i\;and\;j}{Highest\;number\;of\;proximal\;atoms\;between\;amino\;acids\;i\;and\;j}$$

where, any pair of atoms from non-adjacent residues that are within a distance of 4.5 ​Å are considered to be proximal atoms. The highest possible number of proximal atoms between all amino acid types is determined from PSN of all structures in the dataset. The edge-weights obtained for the structural network are stored as an adjacency matrix, which are further used for network comparison. The network images illustrated in this work are drawn on PyMOL (DeLano and Pymol, 2002) using protein cartoon diagrams.

### 2.3 Structure and network comparison

Multiple structures of a protein are subject to pairwise comparison with respect to the backbone structure and their all-atom network. The most common method used for the comparison of protein structures involves measuring the structural divergence between two superimposed atomic coordinates commonly known as the root mean square deviation (RMSD). In this work, the structural divergence between a pair of conformers is calculated as the root mean square deviation of C-alpha atoms, computed using the TM-align tool (Zhang and Skolnick, 2005). The pairwise comparisons result in quantifying the divergence between the backbone conformation of all structures of a protein.

It should be noted that the method of calculating RMSD has several limitations (Li et al., 2020). For instance, in a pair of close to identical structures that vary only in a small random coil or turn regions or a single flexible terminus, the structural comparison can result in a large RMSD. Likewise, a small alteration in the core of the structure or the inter-domain region may significantly impact the resulting structure deviation more greatly than the deviation in loops or terminus. Constant efforts are being made to address these limitations (Kufareva and Abagyan, 2012). On the other hand, graph spectra-based methods that include sidechain orientations consider the change in interactions and global connectivity. This involves the aspect of clustering of nodes and quantifying a match between the clusters. A graph spectra-based network comparison tool termed network dissimilarity score (NDS), mentioned in the introduction section, is employed in this work. This method also quantifies the dissimilarity in the local and global node clustering between a pair of networks. Node clustering represents grouping of nodes with respect to the edges present in the network. Nodes in each cluster (or group) are more connected among themselves compared to that with nodes of other clusters. Changes in local node clusters take place according to changes in edge-weight. In other words, residue grouping changes locally with respect to changes in strength of interactions between residues. These changes in local residue clusters result in overall structural change, which is referred as global clustering change.

An in-house python program is used to compute the NDS between any pair of PSN. The score is calculated by obtaining its three components – EDS, EWCS and CRS. The edge difference score (EDS) directly calculates the difference in their edge weights. The correspondence score (CRS) and eigen value weighted cosine scores (EWCS) are calculated using the spectra of their networks, capturing local and global clustering changes of residues in the PSN respectively. Using these components, NDS is formulated as in Equation (2).

Equation 2 $$NDS=\sqrt{EDS^{2}+EWCS^{2}+{\left(1-CRS\right)}^{2}}$$

The NDS can range from a value of zero, that indicates absolute identical networks, to a value of $\sqrt{3}$ that indicates dissimilarity to the extent of no match between the networks. More details regarding the significance of the components of NDS can be referred from Gadiyaram et al. (2017)

### 2.4 Solvent accessibility and secondary structure based sub-networks

A network that contains a subset of the nodes and edges of the original network makes a sub-network. All PSN are decomposed into sub-networks by choosing specific nodes and edges based on a given criteria. Two types of sub-networks are defined, the first decomposition is based on solvent accessibility of residues and the other is based on secondary structures.

**Fig. 1:**
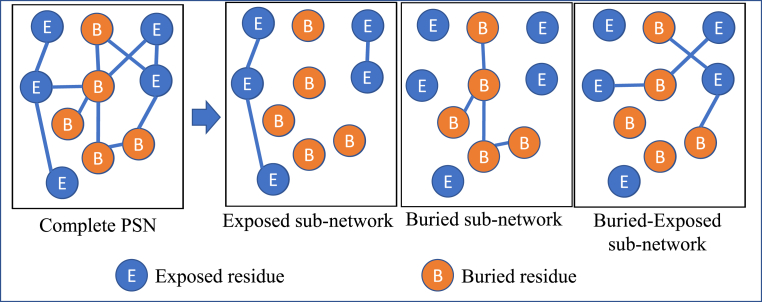
Illustration of decomposing a complete network into sub-networks made of a subset of elements that are selected based on a certain criterion like the solvent accessibility of the corresponding residue in a protein structure network.

*Sub-networks based on solvent accessibility: naccess* tool (Hubbard and Thornton, 1993) is used to compute solvent accessibility of residues in the protein structure. A relative accessible surface area (RSA) threshold of 7% is used in recognising solvent-accessible residues. Residues with RSA lower than 7% are considered as buried in the protein structure. Fig. 1 shows the three sub-networks that are derived for each conformer. E-E: A sub-network with exposed residues as nodes and edges among themselves (solvent-accessible sub-network). B–B: A sub-network with buried residues as nodes and edges among themselves (solvent in-accessible sub-network). B-E: A sub-network that is of bipartite nature such that only edges that connect buried and exposed residues are included. The NDS of sub-networks for all pairs of conformers of a given protein are computed and analysed.

*Sub-networks based on secondary structure: stride* tool (Heinig et al., 2004) is used to assign secondary structures to each residue. Residues that form secondary structures such as helix and strands are considered as ordered residues and the remaining are considered as non-ordered residues. The PSN is decomposed into three sub-networks, similar to sub-networks based on solvent accessibility. O–O: A sub-network of edges between nodes of ordered residues, N–N: A sub-network of edges between nodes of non-ordered residues and O–N: A sub-network of only edges between nodes of an ordered and non-ordered residue. All pairs of sub-networks of a protein are subject to NDS analysis.

### 2.5 Variance of edge weights across an ensemble

The multiple crystal conformers of a protein constitute an ensemble. The mathematical variance in edge-weights describe variation of the spatial proximity and inter-connectivity of corresponding residues with respect to other residues across the ensemble. The edge-weight variance $\left(EV_{ij}\right)$ of each edge is calculated using Equation (3) (analogous to EW-MSF that is discussed in an earlier paper) (Ghosh et al., 2017).

Equation 3 $$EV_{ij}=\frac{1}{N}\overset{N}{\underset{n=1}{\sum }}{\left(I_{n}-\mu \right)}^{2}$$

where, $I_{n}$ is edge-weight of an edge between residues i and j across the ensemble of N structures and μ is the mean of edge-weights across the N structures. The edge-weight variance, thus obtained from Equation (3), quantifies the mean and the fluctuations of all the edges in the network. This metric is used to identify the most variable interactions within the PSN, which points to the regions of protein that show higher variability in network topology.

## 3 Results

In this section, we present our results on the comparison of different conformers of chosen proteins using the conventional parameter RMSD and the parameter network dissimilarity score (NDS) obtained from the global analysis of protein structure networks (PSN). Here, we provide a classification scheme for protein structural domains, based on these analyses.

Crystal conformers of a protein present their native conformational states which have been used for the analysis of their structural variability. A dataset of 56 proteins is assembled with more than five crystal structures for each protein (913 PDB entries) that are of a resolution better than 3 ​Å. PSN for all the crystal conformers are constructed. All pairs of conformers of a protein are subjected to pairwise structural network comparison resulting in 12,251 network dissimilarity scores (NDS). Similarly, Cα-atom root mean square deviation (RMSD) is computed to obtain 12,251 pairwise backbone-structure comparisons. All the computed structure and network comparison scores are listed in Supplementary Table 2 (provided separately).

### 3.1 Structural diversity in individual domain proteins

**Fig. 2:**
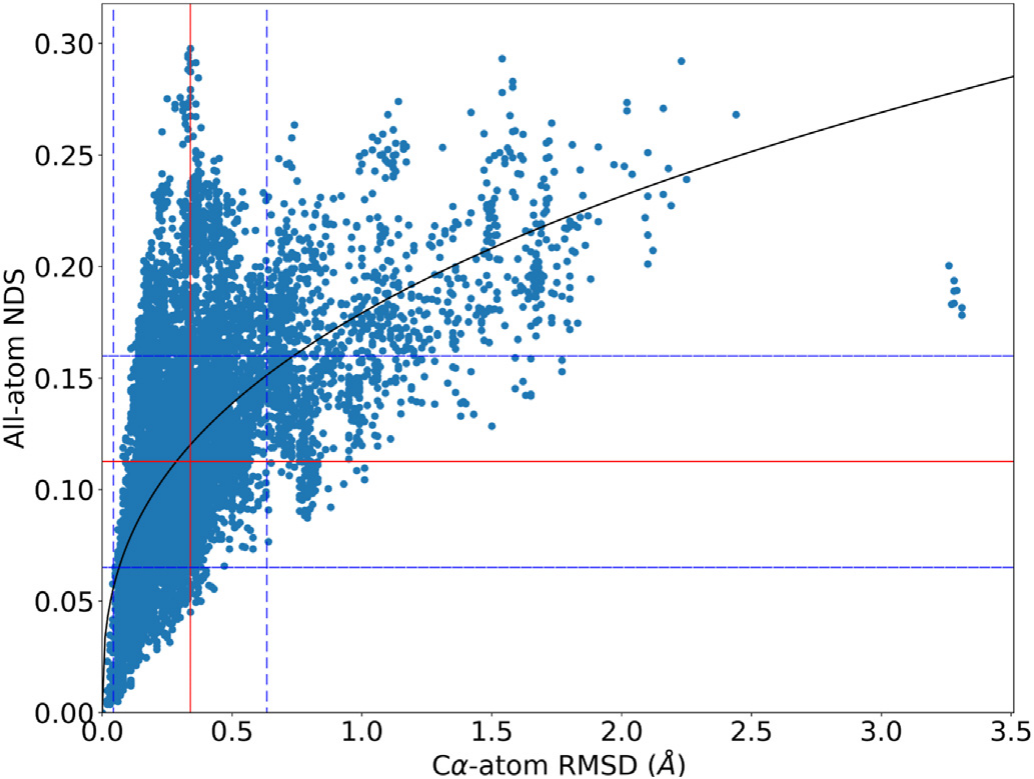
Scatter plot of all pairwise comparisons from the dataset of individual domain proteins. The mean NDS and RMSD of the distribution are shown using the red line and the standard deviation in the distribution is shown using blue dotted lines.

A scatter plot of the comparison scores is illustrated in Fig. 2 where the data points correspond to RMSD on x-axis and NDS on y-axis. Examining the plot, one is provided an understanding of the extent of structural variability and diversity of conformers among individual domain proteins. Mean RMSD for the dataset is found to be 0.34 ​Å with a standard deviation of 0.3 ​Å. Mean NDS is 0.113 with a standard deviation of 0.048, as shown in Fig. 2. From the data presented on the plot of backbone and network comparison scores, a curve of best fit with maximum R-square (R^2^) is plotted. A power equation is found to best fit the data inferring that no linear relationship exists between the RMSD and NDS. The Pearson correlation between the data is found to be 0.59, that supports the understanding that there is no strong linear relationship shared between the scores.

It should be noted that in the scenario where we observe low RMSD scores, NDS is found to vary between a range of small to large values. This implies that, even though there is not much change in the backbone structure of a protein, the variation in sidechain interactions impart a large change into its structural network. However, the converse is not always true. As RMSD increases, NDS has only higher values. This is because variation in backbone will inevitably bring changes to the underlying sidechain interactions.

### 3.2 Characterisation of protein structure variability

The scatter plot is partitioned into four quadrants based on the statistical average that is computed for the entire dataset. The third quadrant, where both RMSD and NDS are lower, corresponds to the comparison of conformers that are highly superposable in terms of backbone and sidechain. Contrarily, both the scores are higher in the first quadrant. If the compared conformers have preserved sidechain interactions but vary only in backbone atom positions, i.e., RMSD is high and NDS is low, the points fall into the fourth quadrant. Also, when the backbone is preserved and there is variation only in sidechain interactions the points fall in the second quadrant.

The data points (scatter) corresponding to individual proteins are analysed. Data points in the third quadrant correspond to conformers with a preserved network and structure and the protein is known to be of rigid type. Likewise, when the scatter spread across other quadrants on the plot, they are classified as non-rigid proteins. Using the scatter from all conformers of each protein, they are grouped into five categories based on the nature of their structural variability:

1. Rigid (R)
2. Preserved network, with variation in backbone (N)
3. Variable network, with preserved backbone (B)
4. Flexible in backbone and network (F)
5. Mixed (M)

**Fig. 3:**
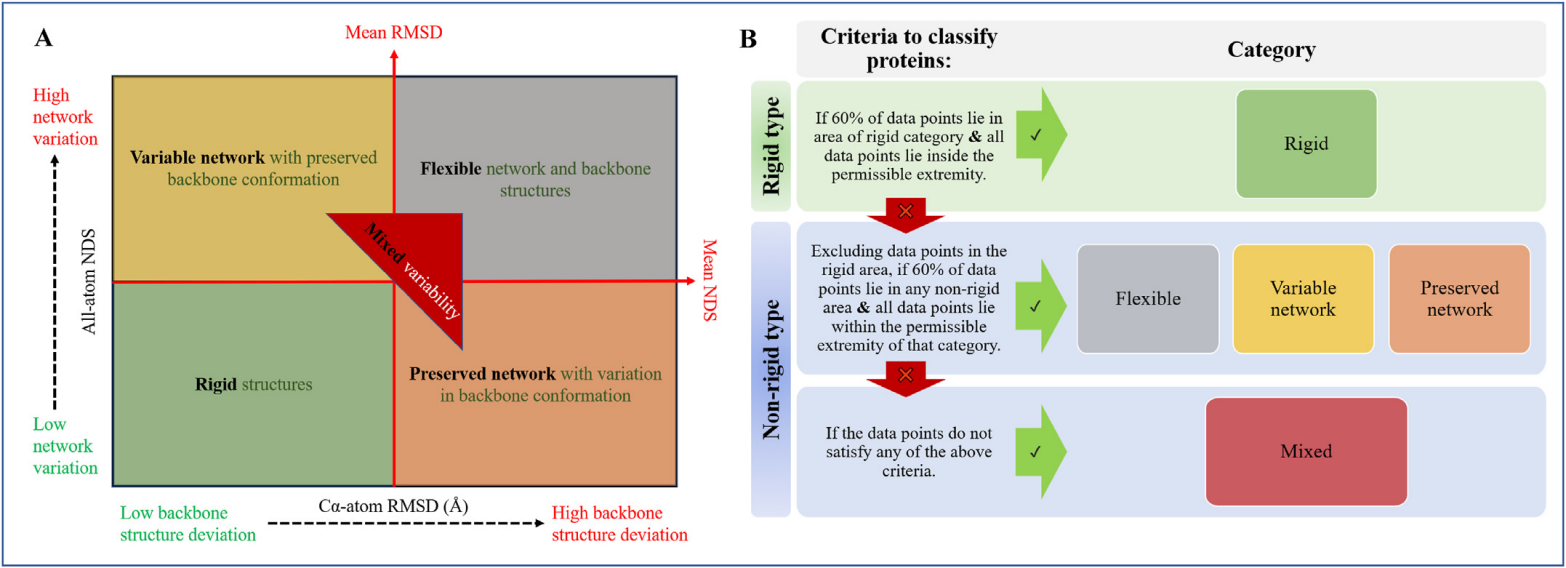
Categorisation of proteins based on the nature of their structural variability. **(A)** A schematic scatter plot that is used to study conformational diversity, where structure deviation information (backbone RMSD) is plot on the x-axis and network dissimilarity (All-atom NDS) is plot on the y-axis, such that the structure variability of a protein would be characterised based on the predominance of datapoints that are localised to specific area designated to each of the categories. **(B)** A flowchart of criteria that is used to characterise the structure variability and categorise proteins in the dataset.

Fig. 3 shows the area on the scatter plot designated for each of the categories. A protein is categorised based on whether more than 60% of its scatter falls within a specific area on the plot that is designated to each of the categories. Also, none of the data points should lie outside a permissible area (The permissible area for each category is discussed along with examples later in this section). Supplementary Fig. 1 shows the percentage of datapoints with lesser than mean NDS and RMSD of the entire dataset. Sixteen proteins are found to have more than or equal to 60% data points in the rigid area (lower than the mean of dataset). However, not all of these proteins satisfy the ‘permissible extremity’ criteria defined for rigid proteins, *i.e.,* all comparison scores of the protein should be lower than the sum of mean and standard deviation of the dataset. Similar criteria are used in the segregation of proteins into each of the categories. The criteria for classifying a protein into each of the categories are discussed in detail with the help of examples.

*Rigid category:* In the discussed scatter plot for each protein, if more than 60% of the scatter lies in the rigid area and none of the data points regress with comparison scores greater than the sum of the mean and standard deviation of the entire dataset, the protein is categorised as a rigid protein. For example, the individual plot of Lysozyme C is shown in Supplementary Figure 2 (A). The comparison of all nine crystal structures of this protein is found to have 94.44% of the scatter in the rigid area and all points within the permissible extremity. The proteins of this category are rigid in nature with conformations of well-preserved backbone and side chain interactions. Listed in Supplementary Table 3 are ten proteins from the dataset that have been categorised as rigid along with the information of mean and standard deviation from their respective distribution of comparison scores. Nearly all proteins have datapoints in this rigid area of the plot which correspond to low conformational variations. Supplementary Fig. 1 shows a bar plot of the percentage of data that lie in the rigid area.

*Preserved network category:* If the interactions (mostly sidechain) in a protein are preserved even when the backbone shows divergence, they are categorised as proteins with preserved network. In the individual scatter plot of such a protein, excluding data points in the rigid area, more than 60% of the scatter lie in the preserved network area that lies on the bottom right of the plot. Also, none of the network comparisons have NDS greater than 0.181 (sum of mean and standard deviation in NDS of the dataset). The nature of structural variability in the protein is a flexible backbone with a preserved network. Four proteins from the dataset, listed in Supplementary Table 4, fall under this category. It should be noted that the backbone deviation in all these four proteins is not significantly high. This may be since the dataset consists of only single-domain proteins. It is possible for a non-single-domain and monomeric protein to have large structural backbone deviation (domain movement) even when the network is well preserved. Such a scenario has been discussed in Ghosh et al. (2017) (refer to Figure 14 of the citation) Supplementary Figure 2 (B) shows the plot of N-acetyltransferase domain-containing protein obtained from their 18 crystal structures as an example. Excluding the data points of this protein in the rigid area, 100% of the scatter is in the preserved network area. All the data points lie within the permissible extremity and hence this protein is of the preserved network category.

*Variable network category:* On the individual scatter plot for each protein, excluding the data points from the rigid area, if more than 60% of the scatter lie in the variable network area and none of the structure comparisons have RMSD greater than 0.64 ​Å (sum of the mean and standard deviation of the entire dataset), the nature of structural variability of these proteins is of a flexible network with a preserved backbone. Eight proteins from the dataset fall under this category which have been summarised in the Supplementary Table 5. In Supplementary Figure 2 (C) we show the individual plot of Prolyl endopeptidase obtained from its twelve crystal structures as an example. Excluding the data points of this protein in the rigid area, 84.62% of the scatter lies in the variable network area. All the points in the plot lie within the permissible extremity and hence the protein is of variable network category.

*Flexible category:* In individual scatter plots, after excluding the data points in the rigid area, if more than 60% of the scatter from a protein has NDS and RMSD greater than the mean of the dataset then the nature of structural variability of these proteins is flexible. Twenty-one proteins from the dataset are found to be flexible and are detailed on Supplementary Table 5. The individual plot of Casein Kinase II (α-subunit) obtained from the eighteen crystal structures is shown as an example in Supplementary Figure 2 (D). Excluding the data points of this protein in the rigid area, 83.55% of the scatter of this protein is found in the flexible area of the plot. Hence this protein is categorised as a flexible protein.

*Mixed category:* Proteins that do not fall into any of the above categories are grouped as mixed category of proteins and the nature of structural variability of the mixed category of proteins is of the non-rigid type. The remaining 13 uncategorised proteins are classified as mixed category and are listed in Supplementary Table 6. Certain proteins that have data points beyond the permissible extremity of comparison scores make it into this category. From the individual plot of Myoglobin *(Equus caballus* shown in Supplementary Figure 2 (E)) it is observed that a lot of the datapoints lie in the preserved network area, however a single datapoint is found to have a significantly higher NDS score, hence this protein is categorised as mixed. Some of the proteins are classified here since one or more conformations diverge drastically from the existing space of conformations and hence the scatter of such a protein do not confine to a specific area in the plot which complicate classifying the protein into any specific category. Similarly, in the case of methionine aminopeptidase (shown in Supplementary Figure 2 (F)) it is discernible than most of the datapoints lie in the variable network area. However, a cluster of data points that depicts backbone structure deviation greater than the mean and standard deviation of the dataset is observed.

**Fig. 4:**
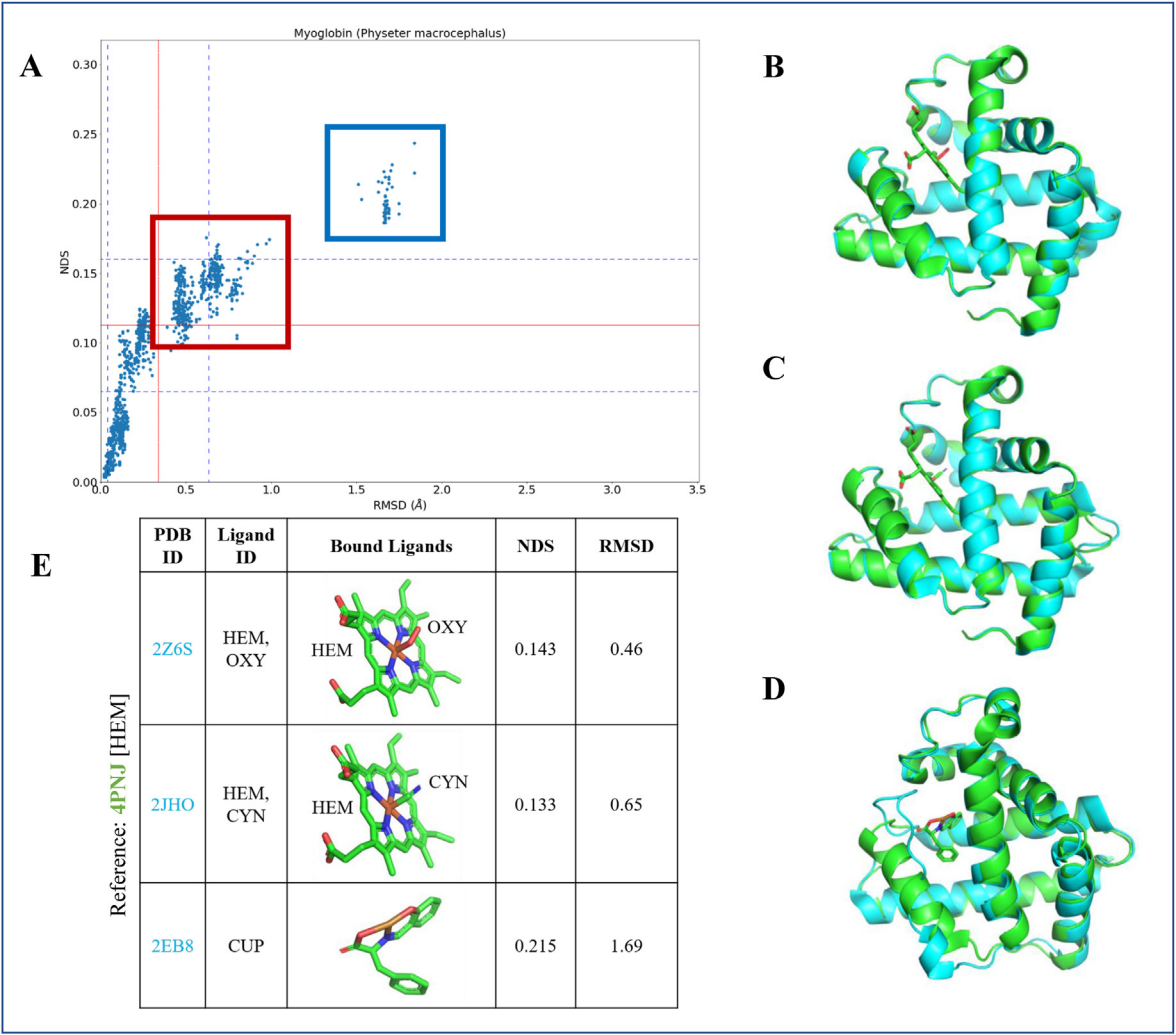
Comparison of different conformational states of a protein can result in clustering of data points observed on their individual scatter plot. **(A)** The plot of comparison scores in the sperm whale myoglobin protein (*Physeter macrocephalus*). Coloured rectangular boxes indicate the clustering of data points on the plot. **(B)** The cartoon diagram of crystal conformer of deoxy conformational state of sperm whale myoglobin that is superposed with the oxy conformational state, where the protein cofactor (HEME) is bound to oxygen is illustrated. The reference deoxy conformational state that is superposed with the protein bound to HEME-CYN **(C)** and a non-HEME bound state **(D)** are also illustrated. **(E)** A table containing information of the comparison scores for the superposed structures and the bound ligands are shown.

If a protein exists in more than one structurally deviant conformational state then the data points corresponding to the comparison appear as more than one cluster on the scatter plot of the individual protein. This is recognised in many non-rigid type proteins and is discernible from the scatter plots shown in Supplementary Fig. 2 (E &F). For example, the myoglobin protein is known to exist in an oxy and deoxy state. In the individual scatter plot of Sperm whale myoglobin (*Physeter Macrocephalus*), distinct clusters are observed as shown in Fig. 4 (A). In the cluster with high comparison scores (illustrated in Fig. 4 (A) using coloured boxes) the compared conformers have diverse topologies and come from different conformational states. For instance, comparing the pair of crystal structures of PDB ID: 4PNJ (deoxy state) and PDB ID: 2Z6S (oxy state) that share a NDS of 0.143 and RMSD of 0.46 ​Å. Likewise, in the comparison of different conformational states of the protein, it is observed that the scatter is spread across different clusters as shown in Fig. 4.

### 3.3 Sub-network analysis

The nature of a residue such as solvent accessibility and secondary structure are frequently conserved during evolution in order to preserve the tertiary structure of the protein and retain its function (Sitbon and Pietrokovski, 2007). The influence of these two parameters on the overall structural variability is presented in this section. To perform this study, the all-atom PSN is decomposed into sub-networks that consist of only specific elements of the network as shown in Fig. 1. The sub-networks with subsets of nodes and edges based on solvent accessibility or secondary structure are analysed.

**Fig. 5:**
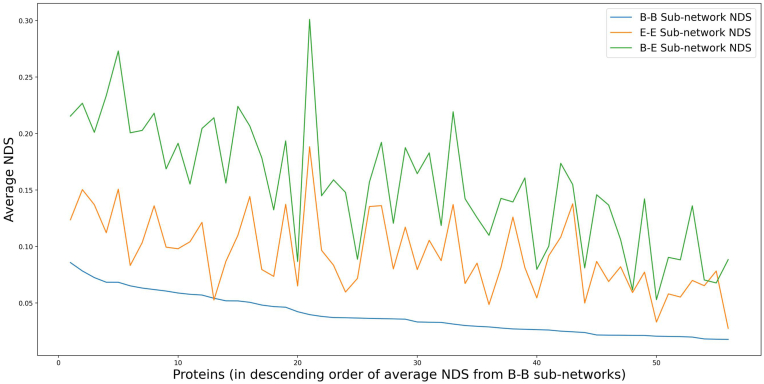
Trace of average NDS from the compared sub-networks, obtained based on solvent accessibility information. The sub-network that captures edges between nodes of only buried residues (B–B) has lower sub-network NDS than the sub-networks that capture edges between nodes of only exposed residues (E–E) and edges between a buried and an exposed residue (B–E). An exception is that of the Rubredoxin protein where it is found that the sub-network of exposed residues (E–E) is well retained.

Using the solvent accessibility information of residues in a protein structure, the all-atom PSN is decomposed into three sub-networks as detailed in the methods section (Section 2.4). The three sub-networks (B–B, E-E and B-E) are subject to pairwise network comparison. All 12,251 pairs of multiple conformers with identical protein sequence from the dataset are compared and the sub-network NDS is computed. The results of sub-network NDS obtained for each protein are illustrated using a boxplot as shown in Supplementary Fig. 3 and the scores are available in Supplementary Table 2. Fig. 5 shows the average NDS of various sub-network comparisons from each protein. It is found that, in almost all protein instances, the sub-network of buried residues (B–B) is more strongly retained than the sub-network of exposed residues (E-E). This is fairly conventional in understanding how the buried residues and the connections amongst themselves are well retained whereas the solvent exposed residues have higher variation amongst their connections. This shows that the mobility of exposed residues contributes to the overall protein structural variability more than the buried residues. Also, it is observed that B-E sub-networks are found to be the most variable. Higher NDS is observed in B-E sub-networks than in E-E sub-networks in all the proteins.

**Fig. 6:**
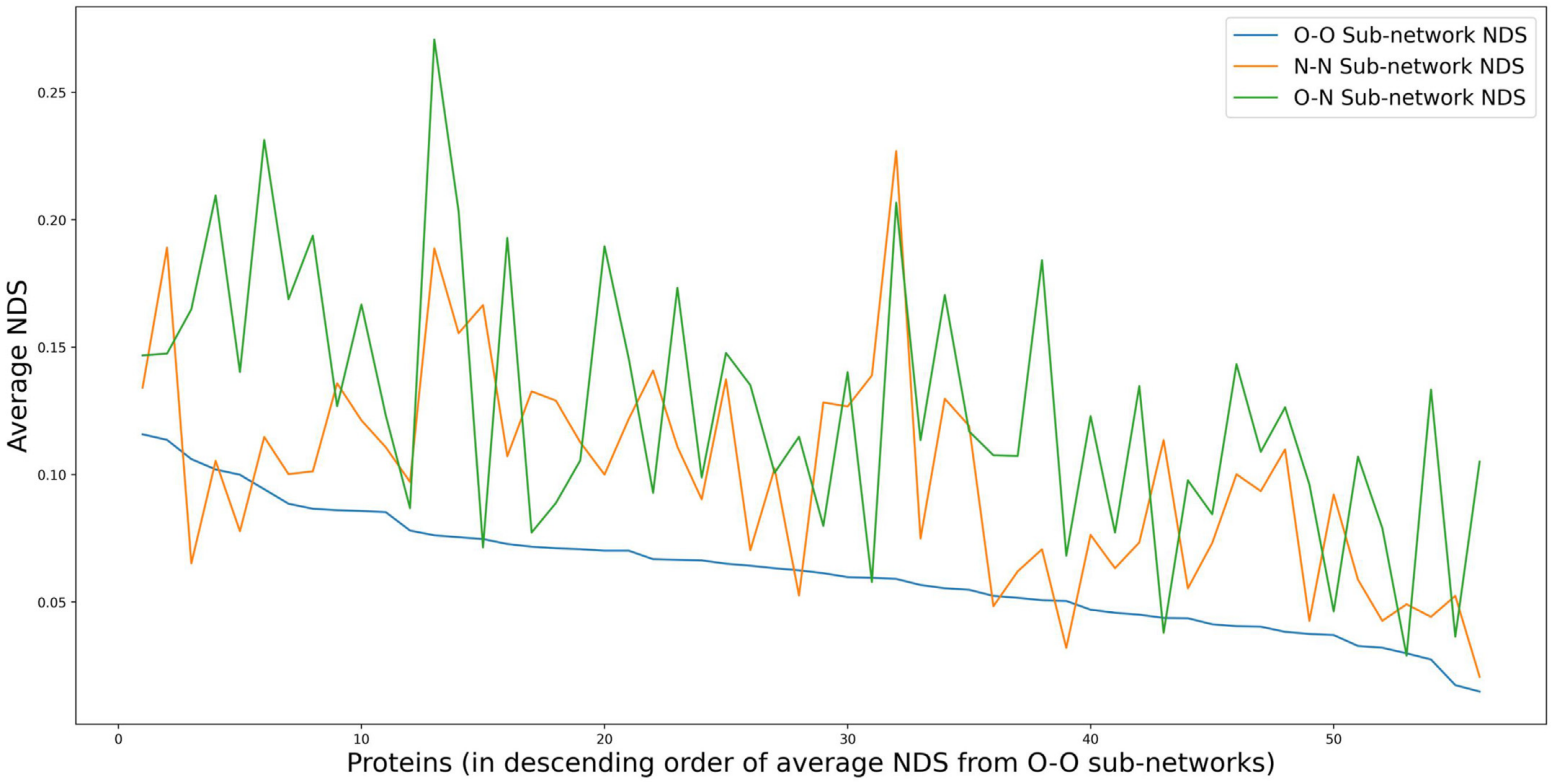
Trace of average NDS, comparing the individual sets of network comparison scores from different kinds of sub-networks that are based on secondary structure information. It is predominantly observed that the sub-network of ordered residues (O–O) is better retained than the sub-network of non-ordered residues (N–N), except in five proteins which are Leucoterine A-4 hydrolase, Quinolate synthase A, S-hydroxynitrile lyase, NADH-cytochrome b5 reductase 3 and rRNA N-glycosidase where they have better retained sub-network of non-ordered residues (N–N). Also, in four other proteins, the sub-network of ordered and non-ordered residues (N–O) is better preserved than sub-network within secondary structures (O–O) which are Heart fatty acid binding protein, Myoglobin (*Physeter macrocephalus*), Peptidyl-tRNA hydrolase and Glutaredoxin.

In a similar kind of analysis, information of secondary structures is used in constructing sub-networks. The residues of a protein are distinguished as ordered and non-ordered residues based on whether they form ordered secondary structures such as helices and sheets or if they make up the non-ordered secondary structures such as coils and turns. Three sub-networks (O–O, N–N and O–N) are generated for each conformer as described in the methods section (Section 2.4). All pairs of sub-networks are compared and the scores are plot on boxplots as shown in Supplementary Fig. 4 and are available in Supplementary Table 2. Fig. 6 shows the average NDS of sub-networks in each protein. In most proteins the sub-networks of non-ordered residues (N–N) have higher dissimilarity than the sub-networks of ordered residues (O–O). This implies that the interactions between non-ordered residues, which are known to be more flexible than ordered residues, have greater variability than the interactions between ordered residues that make up helices and sheets. It is also interesting to note that the O–N sub-network exhibits higher dissimilarity than N–N sub-network in many cases.

### 3.4 Edge-weight variance in protein ensembles

**Fig. 7:**
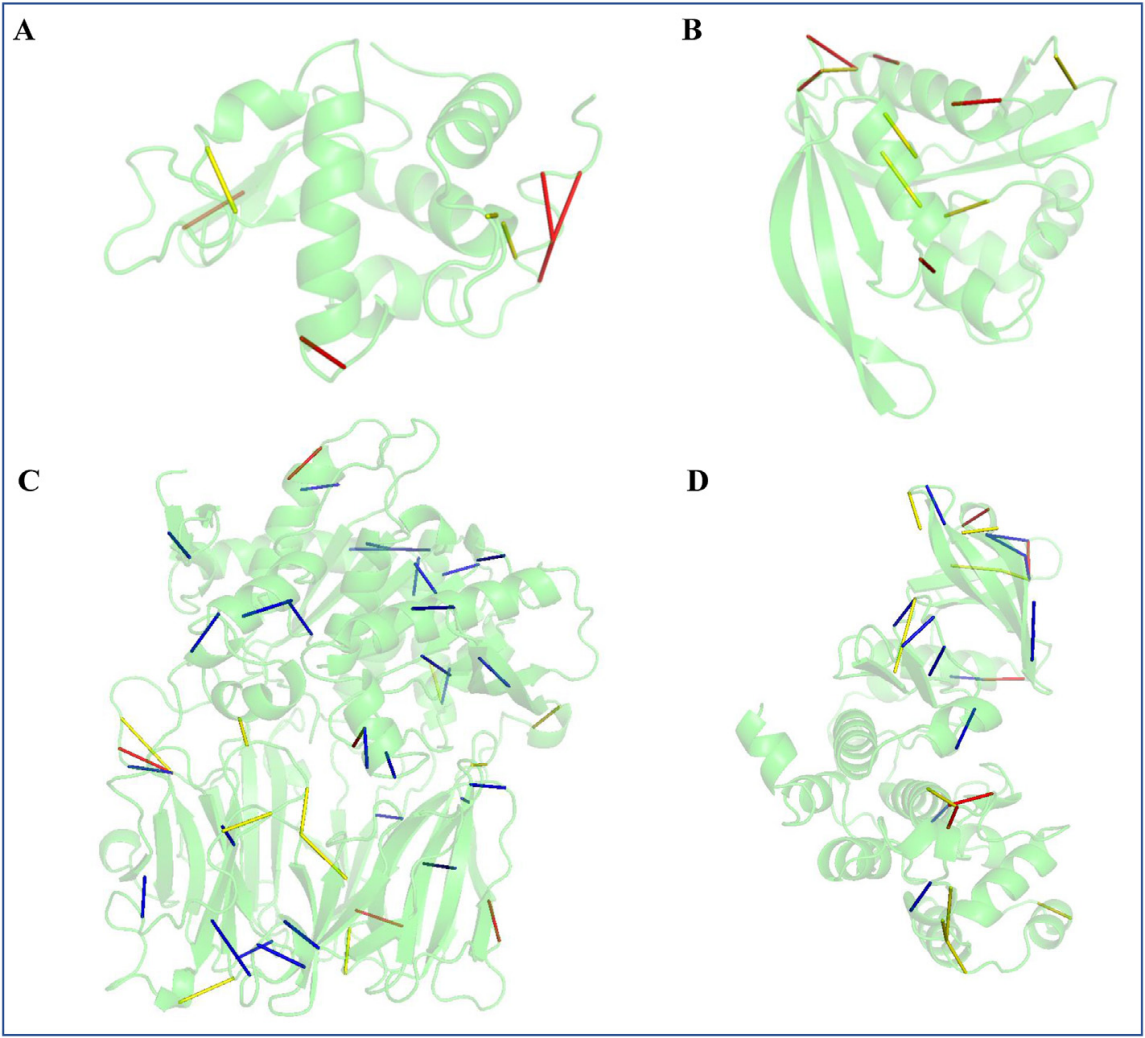
Edge-weight variance profile can describe network variations. Edges of the PSN whose variance in edge-weight is greater than three times their standard deviation are shown in different colours. In descending order of their numerical variance, the top five edges are shown in red, the next ten edges are shown in yellow and the remaining edges, if any are shown in blue. The edge variance profile of **(A)** Lysozyme C **(B)** N-acetyltransferase domain-containing protein, **(C)** Prolyl endopeptidase, **(D)** Casein Kinase II (α-subunit) proteins are shown here.

The conservation of structural interactions within the protein structure is essential for maintaining its function. Consequently, perturbations that alter the intra-connectivity of amino acids can modify the stability (Worth et al., 2011; Pandurangan et al., 2017) or function of the protein (Frauenfelder et al., 2007; Redfern et al., 2008). The spatial proximity between residues in 3D structure of the protein describes the intra-connectivity of residues that are captured using edges in the PSN. Given an ensemble of PSN, variation in proximity of residues is studied by using variance in network edge-weight parameter which is discussed in detail in the methods section. Variance of the edge parameters in every protein of the dataset is computed to yield an edge variance profile. Fig. 7 shows the edges with very high variance in the discussed examples for each category. The coloured edges have a variance greater than three times the standard deviation of the variance recorded in all edges of the given protein. In descending order of the recorded variance, the top five edges are coloured in red, the next ten are coloured in yellow and all the remaining are coloured in blue. The number of such highly variable edges is lower in proteins of rigid and preserved network category whereas they are higher in proteins of network variable and flexible category.

**Fig. 8:**
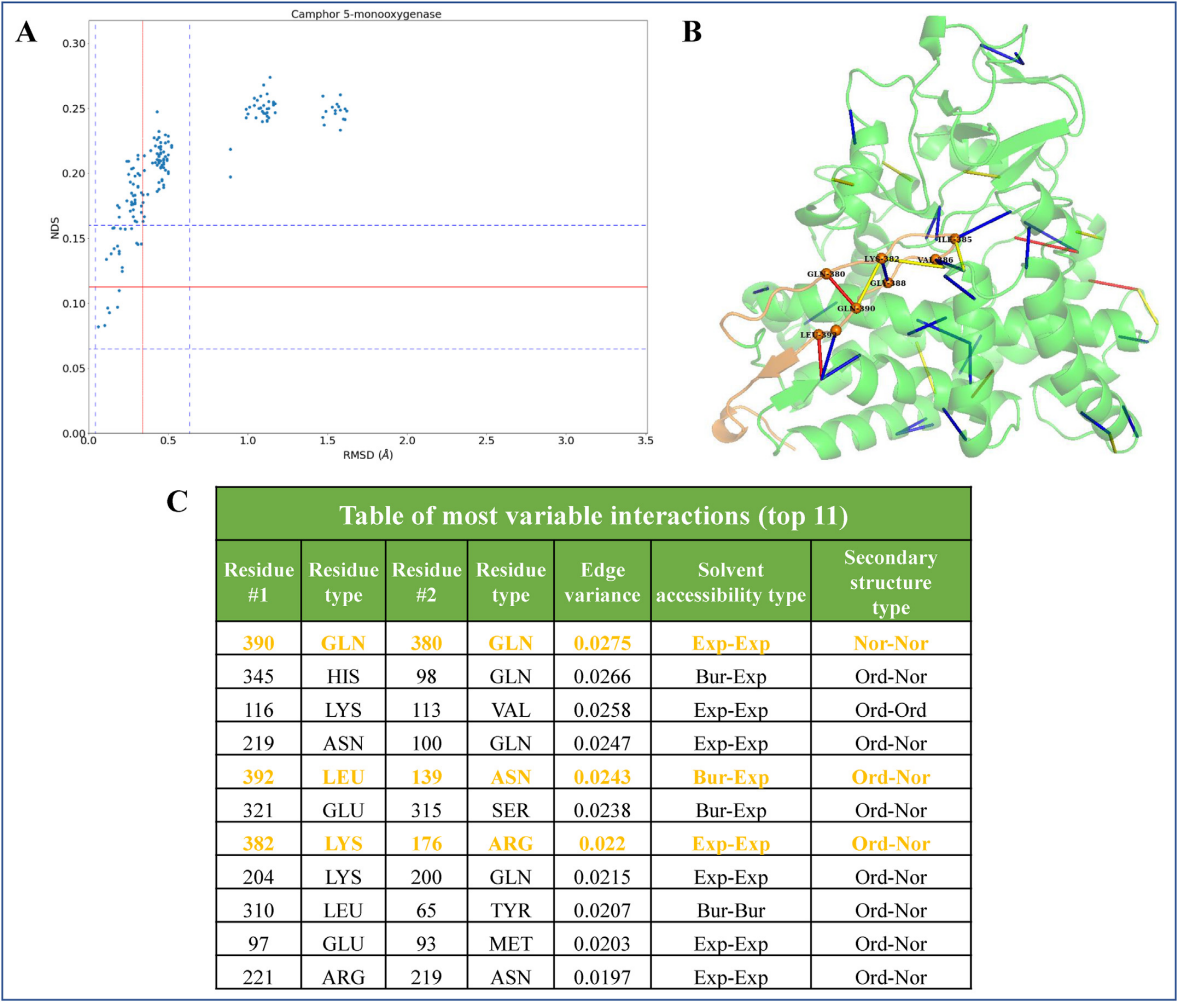
Edge-weight variance in the crystal conformers of Camphor 5- monooxygenase. **(A)** Individual scatter plot of structural comparison of all pair of crystal conformers of Camphor 5-monooxygenase in the dataset. **(B)** Cartoon diagram of the protein structure depicting the edge-weight variance profile, where residues of higher variance are depicted in colours red, yellow and blue in descending order of the numerical variance. The C-terminal region of the protein is coloured in orange to show that most of the variable edges are found to be associated with this region. The Cα atom of nodes that make these variable edges are shown using spheres in the cartoon diagram. **(C)** Table of eleven most variable interactions in the protein. The interactions that are associated with the C-terminal region are highlighted in orange colour.

The method has been discussed with Camphor 5-monooxygenase (Cytochrome P450 protein) as a case study. The data points corresponding to pairwise comparison of all pairs of nineteen crystal structures of Camphor 5-monooxygenase obtained from *Pseudomonas putida* are illustrated on a scatter plot shown in Fig. 8 (A). Since, more than 60% of the scatter of this protein has NDS and RMSD greater than the mean of the dataset, the structural variability of this non-rigid type protein is grouped under the flexible category. In Fig. 8 (A), which shows the individual plot, distinct clusters of the scatter are observed. The first cluster having low RMSD has a diversity of network dissimilarity scores which shows that the structural network of sidechain interactions is variable. The second cluster of data points, with higher NDS and RMSD than the initial cluster, correspond to the comparison of altogether different conformations. In order to identify specific residues and regions of the protein that contribute to such variability we study their edge variance profile.

Fig. 8 (B) shows a cartoon diagram of the structure of camphor 5-monoxygenase where edges of the PSN are coloured based on the edge-weight variance across the ensemble. Residues that are highly variable are identified by arranging the residues in descending order of their edge-weight variance. A list of the eleven most variable interactions (edges of PSN) and their details are shown on the table in Fig. 8 (C). Few of the most variable edges are observed on the C-terminal region that interacts with the core of the protein (shown in Fig. 8 (B) depicted using orange colour). It is inferred from the case that polar residues that are predominantly exposed in the structure have a higher probability of making an edge that is more variable. By performing such an analysis, we make use of the variance profile as a tool to recognise nodes in the PSN that have a greater influence on their overall variability. It will be interesting to analyse the functional relevance of such network variability in our future work.

## 4 Discussion

The flexibility in protein structure enables them to bind to a wide variety of molecules and undergo conformational alterations, to perform its function in living cells. The extent of deviation changes from protein to protein. Native states of proteins that are captured using structure determination methods, such as X-ray diffraction, pave the way in understanding its conformation diversity. The flexibility in atom positions across several conformations of a protein constitutes its structural variability.

The nature of structural variability may vary depending on protein function even when the fold is conserved. Hence, the structural variability of a given protein can be utilized as a metric to correlate with protein structure-function relationship. In this study, proteins have been characterised as rigid or non-rigid based on how diverse their conformers are in terms of their topologies. The mean value along with standard deviation information from the entire dataset is employed in formulating a criterion to segregate the proteins. Some of the salient features emerging from our analyses are presented below.

• Proteins belonging to the family of protein kinase, catalytic subunits (SCOPe family: d.144.1.7) which are Cyclin dependent kinase 2, Casein kinase-II and Mitogen activated protein kinase are all grouped as flexible proteins.
• Orthologs of Myoglobin and dihydrofolate reductase are predominantly characterised as flexible although few are in the ungrouped mixed category.
• Mycocyclosin synthase has network variation with preserved backbone structure, while its homologs NADP nitrous oxide-forming nitric oxide reductase and Camphor 5-monoxygenase are found to be flexible.
• The four members of the fatty acid binding protein family (SCOPe family: b.0.1.2) in the dataset are all grouped in different categories. While the Heart fatty acid binding protein is found to be rigid, Retinol binding protein is flexible. The Liver fatty acid binding protein which is categorised as mixed is observed to have strong network variation. On the other hand, Adipocyte fatty acid binding protein has limited network variation even when there is backbone structure deviation.

It will be interesting to follow a similar protocol in identifying the variability across proteins with difference in sequence such as homologs.

**Fig. 9:**
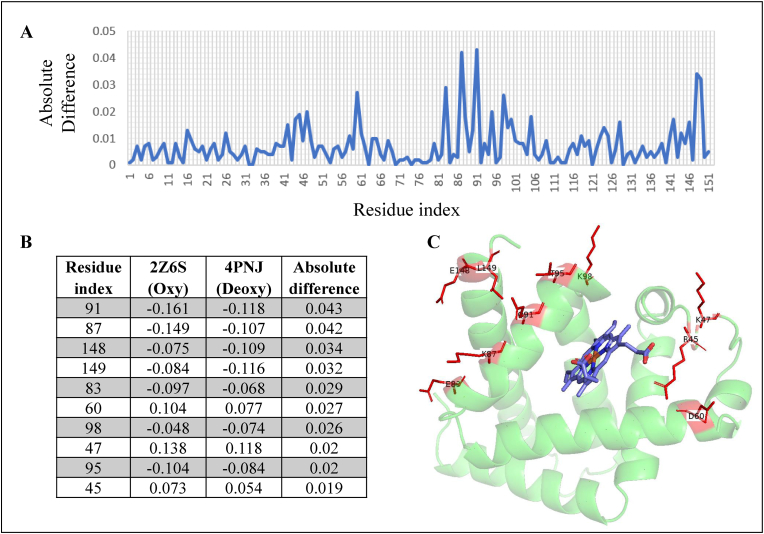
Fiedler vectors analysis between de-oxy state (PDB ID: 4PNJ) and oxy state (PDB ID: 2Z6S) of myoglobin protein. **(A)** Absolute difference between the Fiedler vectors of 4PNJ and 2Z6S. **(B)** Top 10 residues in the structures of myoglobin that show highest absolute difference between Fiedler vectors of 4PNJ and 2Z6S. **(C)** Cartoon diagram of myoglobin protein structure highlighting top 10 residues with highest absolute difference between Fiedler vectors of 4PNJ (de-oxy state) vs 2Z6S (oxy state). These residues are shown as red sticks.

The PSN comparison method used here is shown to be effective in capturing the variation of the overall network. What has not been discussed in detail is how this method is also effective in capturing minute differences in the network such as a change in local or global clustering. The method has been discussed in detail with the help of examples before (Ghosh et al., 2017). We revisit the method by analysing the Fiedler vectors (eigen vector corresponding to second smallest eigen value) between the oxy (PDB ID: 2Z6S) and de-oxy (PDB ID: 4PNJ) states of myoglobin. The highest absolute difference between their Fiedler vectors also correspond to the nodes in the PSN with the most change in local clustering. We identify these residues in the structure of myoglobin protein and find that they are in the vicinity of the HEME cofactor that is known to undergo a structural change between the oxy and deoxy states as shown in Fig. 9.

The analysis of sub-networks based on solvent accessibility show that, in all proteins the sub-network of buried residues (B–B) sub-network, forming the core of the protein is well preserved. It is interesting to note that the average NDS is much higher in the connections between buried and exposed residues (B-E) than in connections between exposed residues (E-E) alone. This may imply that the displacements in exposed residue pairs are more associative than the displacements between buried-exposed connections. Likewise, in the analysis of sub-networks based on secondary structure information, ordered residue connections are found to be substantially well preserved (in forty-seven proteins) as expected. However, the N–N sub-networks and the O–N sub-networks are better preserved than O–O sub-networks in five and four different proteins respectively and the corresponding proteins are listed in the legend for Fig. 6. This implies that in certain scenarios the network of connections between random coils and turns are better preserved than within secondary structures (helices and sheets). The characterization of the variability metric is likely to provide greater insights in complex situations like multidomain that include domain-domain interactions and also across homologs.

Analogous to the edge weight mean square fluctuations (EW-MSF) discussed in reference Ghosh et al. (Ghosh et al., 2017), here we have discussed the edge-weight variance procedure in the context of crystal structure ensembles and their variability. The information of the variance, points to the mobility of nodes in the multiple PSN, in other words, pointing to variability of residue positions in the native structures. Moreover, it helps in understanding the variability among different regions of the same protein. Thus, in addition to quality check programs like PROCHEK (Laskowski et al., 1993), the incorporation of features related to the side-chain conformational preferences (Rose, 2019) and the currently described variability metric, elucidating the dynamics of side-chain interactions, can contribute towards the accuracy enhancement of side chain modelling. The modelled sidechain information can improve the accuracy of CASP and other protein structure prediction methods (Jumper et al., 2021; Leman et al., 2020) that rely on available protein structure information.

## 5 Conclusions

The advantages of studying global and local connectivity within protein structures using graph spectral methods have been exploited in our analysis of structural variations in monomeric proteins. Ensembles of multiple crystal structures of 56 proteins are collected in a dataset for the analysis of structural variability by employing protein structural networks. The conformational diversity is described from pairwise comparison of their backbone structure and network topology that is used to group them into categories based on nature of their structural variability. Most of the proteins in the dataset are categorised as either rigid or flexible conformations. Furthermore, in certain proteins it is observed that the network of edges (mostly sidechain) may be variable even when the backbone positions are preserved, and the vice versa. This is an advantage using a method such as network dissimilarity to study sidechain connectivity rather than just by looking at the backbone structure deviation to understand diversity in protein conformations.

Sub-network analysis reveals that connections of non-ordered secondary structures and solvent exposed residues depict high dissimilarity in inter-residue interactions thus imparting less rigidity to the structure. In a case study, it is seen that edges made with polar residues that are predominantly exposed show greater variability than their counterparts. Such an analysis can also be used as a basis for understanding the variability brought about by external perturbations that may influence the structure and dynamics of a protein.

## Funding statement

VG is supported by CSIR-RA fellowship. Research from NS group is supported by the following agencies or programs of the Government of India: DBT-COE, 10.13039/501100004541Ministry of Human Resource Development, DST-FIST, UGC Center for Advanced Study, Bioinformatics and Computational Biology centre support from 10.13039/501100001407DBT and the DBT-IISc Partnership Program. NS is a J.C. Bose National Fellow. SV is an Honorary Scientist of NASI (National Academy of Sciences, Allahabad, India).

## Author statement

NS conceived the concept and idea for the work SV conceived the concept and idea for the work. VMP took care of all the calculations VG took care of all the calculations. All the authors were involved in formulating the Manuscript.

## Declaration of competing interest

The authors declare that they have no known competing financial interests or personal relationships that could have appeared to influence the work reported in this paper.
