# Supplementary material for: Understanding structural variability in proteins using protein structural networks
Source: Curr Res Struct Biol. 2022 Apr 27;4:134–45. doi: 10.1016/j.crstbi.2022.04.002 (PMC9108755; doi:10.1016/j.crstbi.2022.04.002)
Supplement: Multimedia component 1 [file mmc1.docx]

**Supplementary Information**

*There are a total of 17 pages in this document.*

All figures and tables are labelled as ‘Supplementary’ followed by their captions.

Additional analysis performed are presented in the appendix.

* Table is attached as a separate excel file.

**Contents:**

**Tables**

Supplementary Table 1: Dataset of proteins analysed in this work

Supplementary Table 2: (* Attached seperately, legend included in file)

Supplementary Table 3: Rigid category proteins

Supplementary Table 4: Preserved network with flexible backbone category proteins

Supplementary Table 5: Variable network category proteins

Supplementary Table 6: Flexible category proteins

Supplementary Table 7: Mixed category proteins

**Figures**

Supplementary Figure 1: Rigidity percentage

Supplementary Figure 2: Individual scatter plots from each category

Supplementary Figure 3: Sub-network analysis based on solvent acessibility

Supplementary Figure 4: Sub-network analysis based on secondary structure

**Appendix**

1. Analysis of variability from MD simulations
2. Analysis of variability from NMR structures

**Supplementary Table 1:** The dataset of single-domain single-chain proteins. The 56 proteins that have been studied in this work. The table lists the Uniprot ID, length of each chain and the number of available crystal structures that satisfy our dataset requirements. The table is sorted based on the length of each chain.

| Sl No. | UniProt ID | Chain length | PDB entries | Protein name |
| --- | --- | --- | --- | --- |
| 1 | P23687 | 710 | 12 | Prolyl endopeptidase |
| 2 | P09960 | 611 | 43 | Leukotriene A-4 hydrolase |
| 3 | Q40577 | 548 | 16 | 5-epi-aristolochene synthase |
| 4 | Q47PU3 | 542 | 6 | Phenylacetone monooxygenase |
| 5 | P0A6D3 | 427 | 7 | 3-phosphoshikimate 1-carboxyvinyltransferase |
| 6 | P00183 | 415 | 19 | Camphor 5-monooxygenase |
| 7 | P23295 | 403 | 12 | NADP nitrous oxide-forming nitric oxide reductase |
| 8 | P9WPP7 | 396 | 21 | Mycocyclosin synthase |
| 9 | P71278 | 365 | 18 | Pentaerythritol tetranitrate reductase |
| 10 | Q16539 | 360 | 24 | Mitogen-activated protein kinase 14 |
| 11 | P04547 | 351 | 8 | DNA beta-glucosyltransferase |
| 12 | P00636 | 338 | 11 | Fructose-1,6-bisphosphatase 1 |
| 13 | P28523 | 332 | 18 | Casein kinase II (alpha subunit) |
| 14 | P05326 | 331 | 36 | Isopenicillin N synthase |
| 15 | P15121 | 316 | 9 | Aldo-keto reductase family 1 member B1 |
| 16 | P32396 | 310 | 9 | Coproporphyrin III ferrochelatase |
| 17 | P56588 | 302 | 8 | Endo-1,4-beta-xylanase |
| 18 | O57767 | 300 | 7 | Quinolinate synthase A |
| 19 | P24941 | 298 | 8 | Cyclin-dependent kinase 2 |
| 20 | D4Z2G1 | 296 | 7 | Haloalkane dehalogenase |
| 21 | Q3UZZ6 | 295 | 7 | Sulfotransferase 1 family member D1 |
| 22 | O00625 | 290 | 6 | Pirin |
| 23 | P83686 | 272 | 8 | NADH-cytochrome b5 reductase 3 |
| 24 | P0AE18 | 264 | 22 | Methionine aminopeptidase |
| 25 | P00918 | 260 | 38 | Carbonic anhydrase 2 |
| 26 | P52704 | 257 | 6 | (S)-hydroxynitrile lyase |
| 27 | D9J2T9 | 246 | 58 | rRNA N-glycosidase |
| 28 | P74325 | 244 | 9 | Sucrose-phosphate phosphatase |
| 29 | P16184 | 206 | 11 | Dihydrofolate reductase (Pneumocystis carinii) |
| 30 | P21816 | 200 | 23 | Cysteine dioxygenase type 1 |
| 31 | P20425 | 195 | 6 | UMP-CMP kinase |
| 32 | D0C9L6 | 193 | 10 | Peptidyl-tRNA hydrolase |
| 33 | P00374 | 187 | 37 | Dihydrofolate reductase (Homo sapiens) |
| 34 | P9WPY3 | 176 | 14 | Shikimate kinase |
| 35 | P0A6K3 | 169 | 6 | Peptide deformylase |
| 36 | P62937 | 165 | 54 | Peptidyl-prolyl cis-trans isomerase A |
| 37 | P15494 | 160 | 10 | Major pollen allergen Bet v 1-A |
| 38 | Q9HV14 | 160 | 18 | N-acetyltransferase domain-containing protein |
| 39 | P0A017 | 159 | 20 | Dihydrofolate reductase (Staphylococcus aureus) |
| 40 | P0ABQ4 | 159 | 9 | Dihydrofolate reductase (Escherichia coli) |
| 41 | P26281 | 159 | 19 | 2-amino-4-hydroxy-6-hydroxymethyldihydropteridine pyrophosphokinase |
| 42 | P24666 | 158 | 6 | Low molecular weight phosphotyrosine protein phosphatase |
| 43 | P68082 | 154 | 59 | Myoglobin (Equus caballus) |
| 44 | P02185 | 154 | 47 | Myoglobin (Physeter macrocephalus) |
| 45 | Q7M422 | 152 | 9 | Hemoglobin V |
| 46 | P00698 | 147 | 9 | Lysozyme C |
| 47 | O96438 | 146 | 6 | Tryparedoxin |
| 48 | H3JQV6 | 146 | 6 | Myoglobin (Thunnus atlanticus) |
| 49 | P09455 | 135 | 7 | Retinol-binding protein 1 |
| 50 | P05413 | 133 | 8 | Heart Fatty acid-binding protein |
| 51 | P15090 | 132 | 17 | Adipocyte Fatty acid-binding protein |
| 52 | P07148 | 127 | 10 | Liver Fatty acid-binding protein |
| 53 | P16113 | 125 | 17 | Photoactive yellow protein |
| 54 | Q9NLB2 | 111 | 6 | Glutaredoxin |
| 55 | P80176 | 83 | 8 | High-potential iron-sulfur protein |
| 56 | P24297 | 54 | 8 | Rubredoxin |

**Supplementary Table 3:** From the individual scatter plot of the computed comparison scores ten proteins are found to be classified as rigid in the dataset of individual domain proteins. The table shows the detailed list of rigid category proteins in the dataset along with the statistical information of the mean and standard deviation in each protein.

| **UniProt ID** | **Protein Name** | **Chain Length** | **#Structures** | **Mean ± SD RMSD (Å)** | **Mean ± SD NDS** |
| --- | --- | --- | --- | --- | --- |
| P80176 | High-potential iron-sulfur protein | 83 | 8 | 0.098 ± 0.047 | 0.025 ± 0.017 |
| P24297 | Rubredoxin | 54 | 8 | 0.12 ± 0.058 | 0.046 ± 0.032 |
| P56588 | Endo-1,4-beta-xylanase | 302 | 8 | 0.12 ± 0.023 | 0.069 ± 0.015 |
| Q9NLB2 | Glutaredoxin | 111 | 6 | 0.121 ± 0.04 | 0.037 ± 0.011 |
| P52704 | (S)-hydroxynitrile lyase | 257 | 6 | 0.143 ± 0.05 | 0.106 ± 0.022 |
| P05413 | Heart Fatty acid-binding protein | 133 | 8 | 0.171 ± 0.059 | 0.056 ± 0.014 |
| P21816 | Cysteine dioxygenase type 1 | 200 | 23 | 0.23 ± 0.08 | 0.091 ± 0.024 |
| P00698 | Lysozyme C | 147 | 9 | 0.252 ± 0.096 | 0.067 ± 0.025 |
| P62937 | Peptidyl-prolyl cis-trans isomerase A | 165 | 54 | 0.279 ± 0.111 | 0.085 ± 0.021 |
| Q7M422 | Hemoglobin V | 152 | 9 | 0.279 ± 0.142 | 0.074 ± 0.022 |

**Supplementary Table 4:** Four proteins from the dataset of individual domain proteins are found to be grouped under the preserved network category. The mean and standard deviation in each protein is listed.

| **UniProt ID** | **Protein Name** | **Chain Length** | **#Structures** | **Mean ± SD RMSD (Å)** | **Mean ± SD NDS** |
| --- | --- | --- | --- | --- | --- |
| Q9HV14 | N-acetyltransferase domain-containing protein | 160 | 18 | 0.303 ± 0.11 | 0.054 ± 0.016 |
| P15090 | Adipocyte Fatty acid-binding protein | 132 | 17 | 0.326 ± 0.11 | 0.08 ± 0.02 |
| O96438 | Tryparedoxin | 146 | 6 | 0.368 ± 0.149 | 0.079 ± 0.028 |
| P24666 | Low molecular weight phosphotyrosine protein phosphatase | 158 | 6 | 0.368 ± 0.099 | 0.084 ± 0.017 |

**Supplementary Table 5:** The list of proteins from the variable network category in the dataset. The mean and standard deviation in each of the eight proteins are detailed in the table. These proteins do not have much variability in their backbone, although, high variability is observed in sidechain connectivity.

| **UniProt ID** | **Protein Name** | **Chain Length** | **#Structures** | **Mean ± SD RMSD (Å)** | **Mean ± SD NDS** |
| --- | --- | --- | --- | --- | --- |
| P9WPP7 | Mycocyclosin synthase | 396 | 21 | 0.193 ± 0.054 | 0.11 ± 0.02 |
| D9J2T9 | rRNA N-glycosidase | 246 | 58 | 0.204 ± 0.086 | 0.093 ± 0.025 |
| P23687 | Prolyl endopeptidase | 710 | 12 | 0.234 ± 0.086 | 0.214 ± 0.059 |
| P09960 | Leukotriene A-4 hydrolase | 611 | 43 | 0.252 ± 0.104 | 0.181 ± 0.031 |
| P71278 | Pentaerythritol tetranitrate reductase | 365 | 18 | 0.252 ± 0.069 | 0.118 ± 0.02 |
| P0A6D3 | 3-phosphoshikimate 1-carboxyvinyltransferase | 427 | 7 | 0.254 ± 0.135 | 0.136 ± 0.023 |
| P00636 | Fructose-1,6-bisphosphatase 1 | 338 | 11 | 0.267 ± 0.067 | 0.143 ± 0.027 |
| D4Z2G1 | Haloalkane dehalogenase | 296 | 7 | 0.294 ± 0.128 | 0.132 ± 0.036 |

**Supplementary Table 6:** Most proteins from the dataset of individual domain proteins are grouped as flexible category proteins based on the nature of their structural variability. The list of twenty-one proteins of this category along with their statistical mean and standard deviation is listed.

| **UniProt ID** | **Protein Name** | **Chain Length** | **#Structures** | **Mean ± SD RMSD (Å)** | **Mean ± SD NDS** |
| --- | --- | --- | --- | --- | --- |
| P23295 | NADP nitrous oxide-forming nitric oxide reductase | 403 | 12 | 0.333 ± 0.127 | 0.144 ± 0.034 |
| O00625 | Pirin | 290 | 6 | 0.381 ± 0.115 | 0.166 ± 0.025 |
| D0C9L6 | Peptidyl-tRNA hydrolase | 193 | 10 | 0.386 ± 0.198 | 0.094 ± 0.038 |
| P02185 | Myoglobin (*Physeter macrocephalus*) | 154 | 47 | 0.387 ± 0.357 | 0.097 ± 0.052 |
| P32396 | Coproporphyrin III ferrochelatase | 310 | 9 | 0.388 ± 0.199 | 0.146 ± 0.027 |
| P15121 | Aldo-keto reductase family 1 member B1 | 316 | 9 | 0.426 ± 0.202 | 0.148 ± 0.059 |
| P00374 | Dihydrofolate reductase (*Homo sapiens*) | 187 | 37 | 0.49 ± 0.215 | 0.147 ± 0.032 |
| H3JQV6 | Myoglobin (*Thunnus atlanticus*) | 146 | 6 | 0.521 ± 0.398 | 0.096 ± 0.027 |
| P28523 | Casein kinase II (alpha subunit) | 332 | 18 | 0.529 ± 0.188 | 0.172 ± 0.024 |
| P09455 | Retinol-binding protein 1 | 135 | 7 | 0.591 ± 0.329 | 0.134 ± 0.043 |
| P00183 | Camphor 5-monooxygenase | 415 | 19 | 0.608 ± 0.433 | 0.206 ± 0.04 |
| O57767 | Quinolinate synthase A | 300 | 7 | 0.66 ± 0.421 | 0.166 ± 0.053 |
| P83686 | NADH-cytochrome b5 reductase 3 | 272 | 8 | 0.666 ± 0.41 | 0.148 ± 0.066 |
| P16184 | Dihydrofolate reductase (*Pneumocystis carinii*) | 206 | 11 | 0.731 ± 0.179 | 0.16 ± 0.037 |
| P0ABQ4 | Dihydrofolate reductase (*Escherichia coli*) | 159 | 9 | 0.742 ± 0.376 | 0.131 ± 0.042 |
| P26281 | 2-amino-4-hydroxy-6-hydroxymethyldihydropteridine pyrophosphokinase | 159 | 19 | 0.864 ± 0.583 | 0.141 ± 0.068 |
| P74325 | Sucrose-phosphate phosphatase | 244 | 9 | 0.878 ± 1.306 | 0.116 ± 0.042 |
| P04547 | DNA beta-glucosyltransferase | 351 | 8 | 0.915 ± 0.704 | 0.183 ± 0.031 |
| P9WPY3 | Shikimate kinase | 176 | 14 | 1.056 ± 0.435 | 0.139 ± 0.031 |
| Q16539 | Mitogen-activated protein kinase 14 | 360 | 24 | 1.104 ± 0.393 | 0.174 ± 0.026 |
| P24941 | Cyclin-dependent kinase 2 | 298 | 8 | 1.62 ± 0.549 | 0.235 ± 0.036 |

**Supplementary Table 7:** The list of thirteen proteins from the dataset that are grouped under the mixed category of individual domain proteins based on the nature of their structural variability. The mean and standard deviation of the computed comparison scores in each protein is presented.

| **UniProt ID** | **Protein Name** | **Chain Length** | **#Structures** | **Mean ± SD RMSD (Å)** | **Mean ± SD NDS** |
| --- | --- | --- | --- | --- | --- |
| P05326 | Isopenicillin N synthase | 331 | 36 | 0.22 ± 0.097 | 0.109 ± 0.027 |
| P0A017 | Dihydrofolate reductase (*Staphylococcus aureus*) | 159 | 20 | 0.221 ± 0.099 | 0.087 ± 0.031 |
| Q3UZZ6 | Sulfotransferase 1 family member D1 | 295 | 7 | 0.26 ± 0.1 | 0.116 ± 0.024 |
| P00918 | Carbonic anhydrase 2 | 260 | 38 | 0.264 ± 0.124 | 0.116 ± 0.029 |
| P68082 | Myoglobin (*Equus caballus*) | 154 | 59 | 0.291 ± 0.228 | 0.086 ± 0.026 |
| P0AE18 | Methionine aminopeptidase | 264 | 22 | 0.296 ± 0.136 | 0.131 ± 0.029 |
| P07148 | Liver Fatty acid-binding protein | 127 | 10 | 0.314 ± 0.141 | 0.084 ± 0.033 |
| P16113 | Photoactive yellow protein | 125 | 17 | 0.327 ± 0.171 | 0.102 ± 0.024 |
| P0A6K3 | Peptide deformylase | 169 | 6 | 0.38 ± 0.166 | 0.099 ± 0.041 |
| Q40577 | 5-epi-aristolochene synthase | 548 | 16 | 0.417 ± 0.21 | 0.173 ± 0.024 |
| P20425 | UMP-CMP kinase | 195 | 6 | 0.437 ± 0.278 | 0.122 ± 0.028 |
| P15494 | Major pollen allergen Bet v 1-A | 160 | 10 | 0.454 ± 0.262 | 0.092 ± 0.028 |
| Q47PU3 | Phenylacetone monooxygenase | 542 | 6 | 0.693 ± 0.636 | 0.222 ± 0.045 |

**Supplementary Figure 1:** Percentage of datapoints that are within the rigid area of individual scatter plots of all proteins of the dataset. When the NDS and RMSD value for a pairwise comparision is below the value of the mean of the dataset then the datapoint would lie in the rigid area. The percentage of datapoints from each protein in our dataset that lie within this area is plot here.


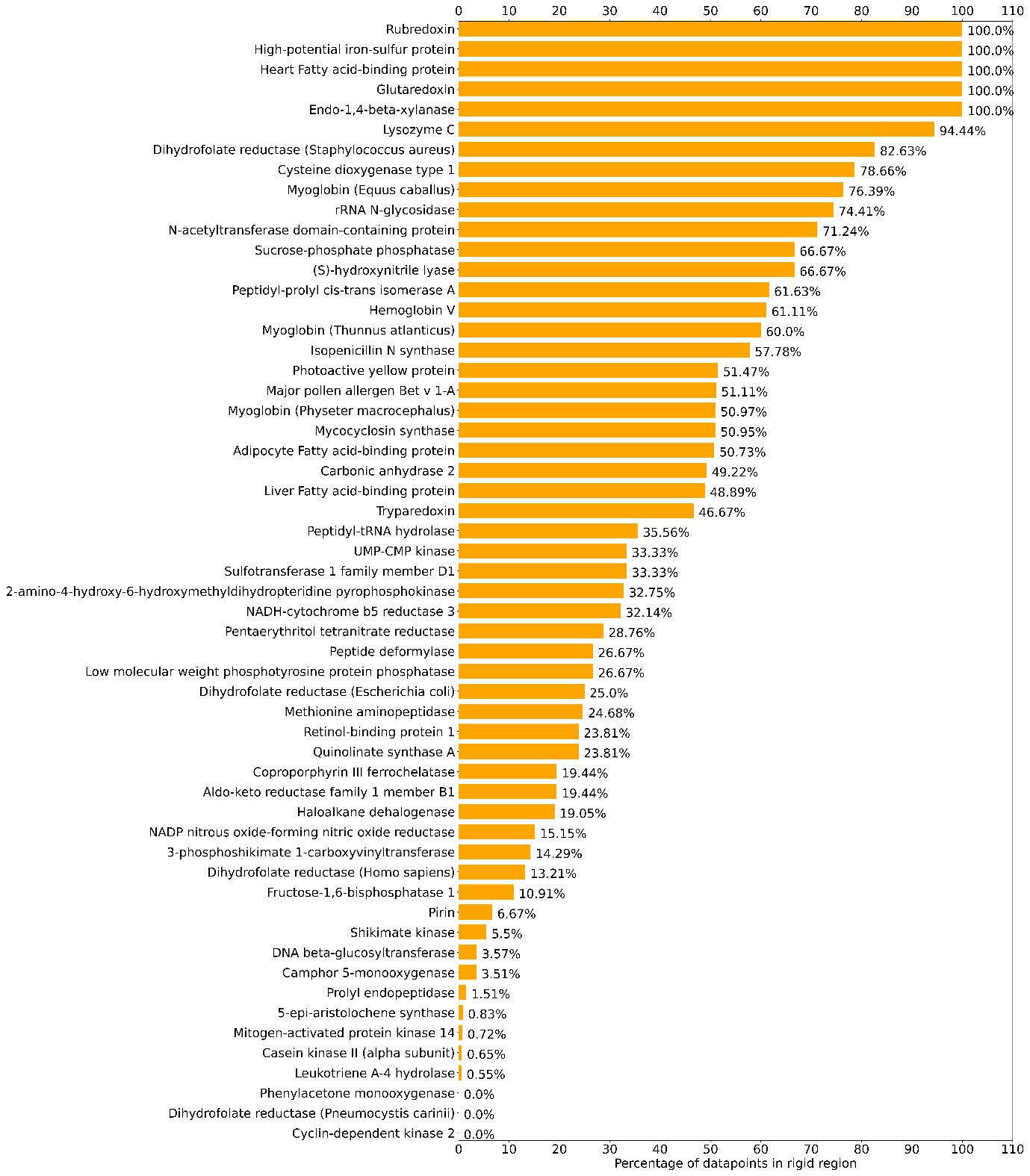


**Supplementary Figure 2:** Individual scatter plots from each category.

**(A)** Individual plot of Lysozyme C and the criterion for rigid category of proteins. When the conformers of a protein have rigid backbone and network topologies, the protein is categorised as a rigid protein. More than 60% of the scatter from rigid category protein should lie in the rigid area and all the data points should lie within the permissible extremity.

**(B)** Individual plot of N-acetyltransferase domain-containing proteins and the criterion for preserved network category of proteins. Proteins with a flexible backbone (albeit lower network dissimilarity) are categorised as preserved network proteins. In the proteins of this category, 60% of the scatters on the plot excluding those in the rigid area lie in the preserved network area and all the data points lie within the permissible extremity.

**(C)** Individual scatter plot of Prolyl endopeptidase and the criterion for variable network category of proteins. Conformers of a protein with dissimilar networks but a preserved backbone are categorised as variable network proteins. More than 60% of the scatter excluding those in the rigid area lie in the network variable area and all the data points are within the permissible extremity.

**(D)** Individual scatter plot of Casein Kinase II (α-subunit) and the criterion for flexible category of proteins. Conformers of a protein that have high dissimilarity in network and backbone is categorised as a flexible protein. For a protein to be categorised as flexible, more than 60% of their scatter excluding those in the rigid area should lie within the flexible area. Any remaining data points can lie anywhere across the map and yet be classified as flexible category protein.

Individual scatter plots of **(E)** Myoglobin (*Equus caballus*) shows that the backbone structure is varying with small alterations in network. However, since more than 60% of the datapoints do not lie in area of any specific category they are classified as mixed category protein. The plot of **(F)** Methionine aminopeptidase has dissimilar network with preserved backbone. However, a cluster of datapoints having RMSD greater than the mean and standard deviation of the dataset are observed. Since this does not satisfy the criteria that datapoints should also lie within this permissible extremity of this category, the protein is grouped under the mixed category. The individual plots of proteins that show more than one cluster of the scatter can correspond to the comparison of different conformational states which is clearly observable in these cases.


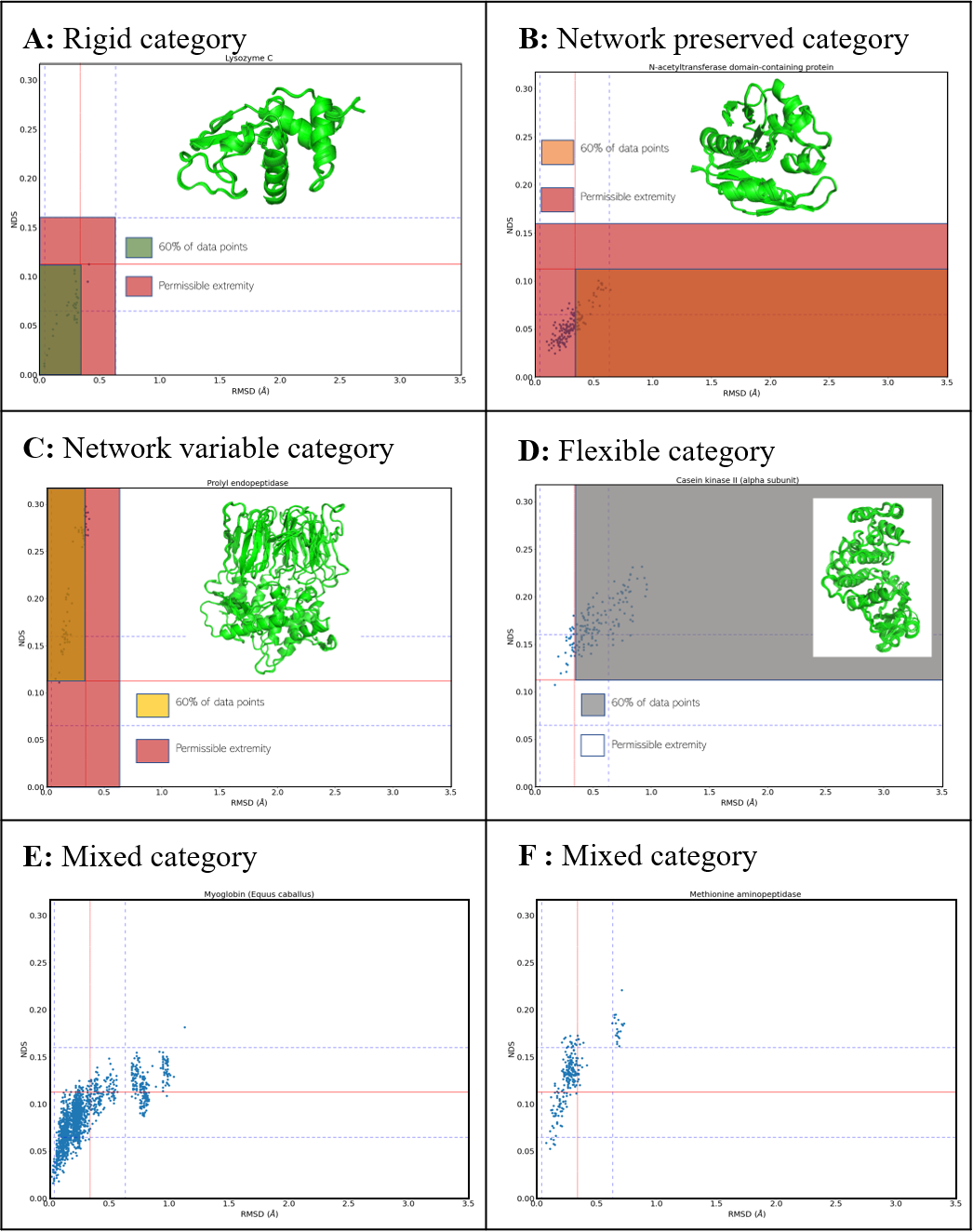


**Supplementary Figure 3:** Sub-network analysis based on solvent acessibility. Sub-networks are generated for each crystal conformer and NDS from comparision of all pairs are computed. The blue, orange and green boxplot drawn for each proetin shows the distribution of NDS from sub-network of buried residues, sub-network of exposed residues and sub-network of buried-exposed residues respectively. It is observed that on an average, the sub-network of interactions between buried residues (B-B) shows much less variability than the sub-network of interactions between exposed residues (E-E) which in turn have lower variability than sub-network of interactions between buried and exposed residues (B-E). An exception is the small 54 residue Rubredoxin protein which is composed of only 7 buried residues while the structure is predominantly exposed.


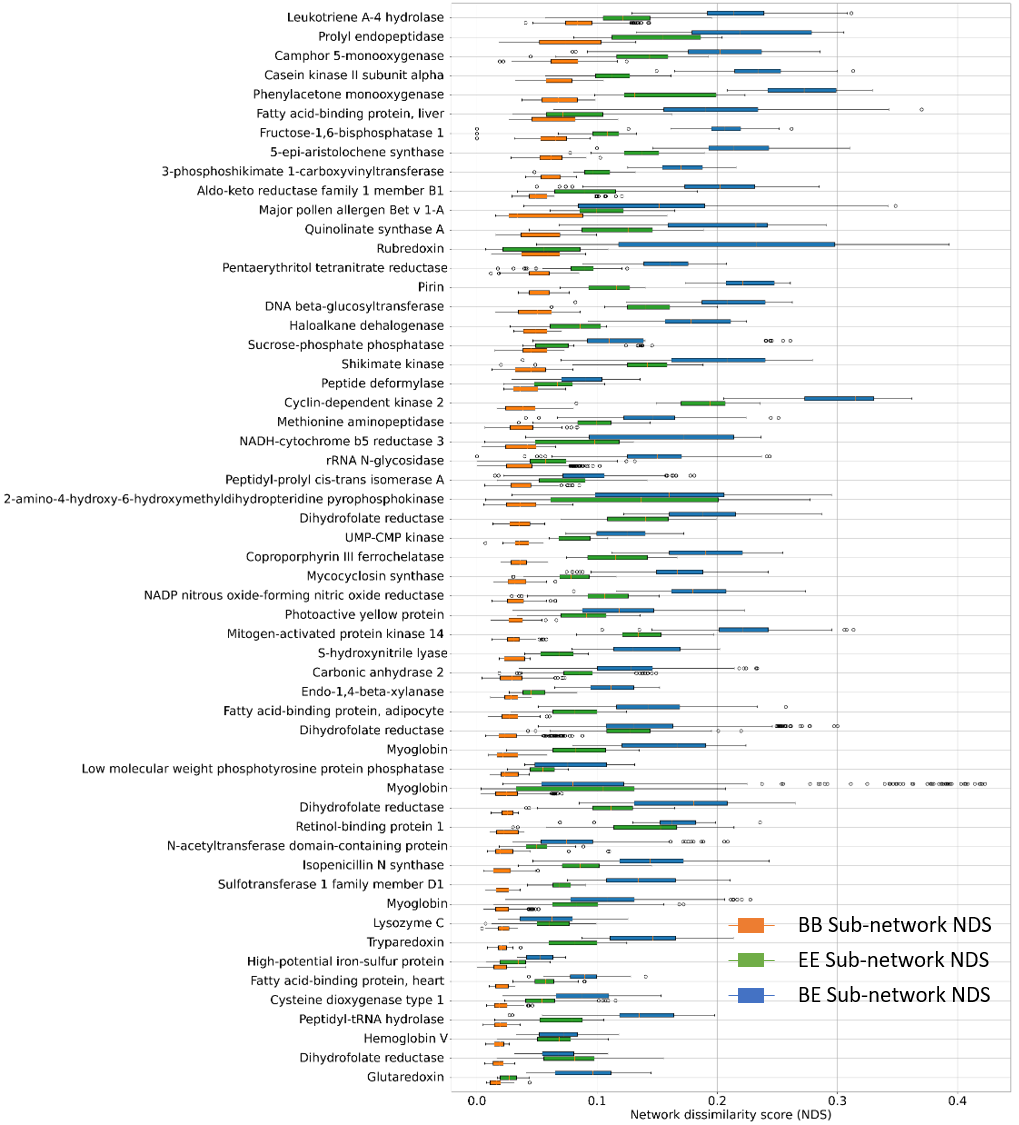


**Supplementary Figure 4:** Sub-network analysis based on secondary structure. NDS is calculated for all pair of sub-networks from a given protein. The boxplot in blue color represents the distribution of NDS in the comparision of sub-netwroks of non-ordered residues. The boxplot in orange shows the distribution of NDS for all pair of sub-networks based on ordered residues. It is observed that, most often, sub-networks of non-ordered residues have higher average dissimilarity than dissimilarity in ordered sub-networks which suggests that the sub-network of non-ordered residues strongly influence the overall variability of the protein structure. However, in proteins like Leucoterine A-4 hydrolase, Quinolate synthase A, S-hydroxynitrile lyase, NADH-cytochrome b5 reductase 3 and rRNA N-glycosidase, the average dissimilarity in sub-network of non-ordered residues is lower. Majority of the residues in these proteins are ordered in nature forming helices and sheets.


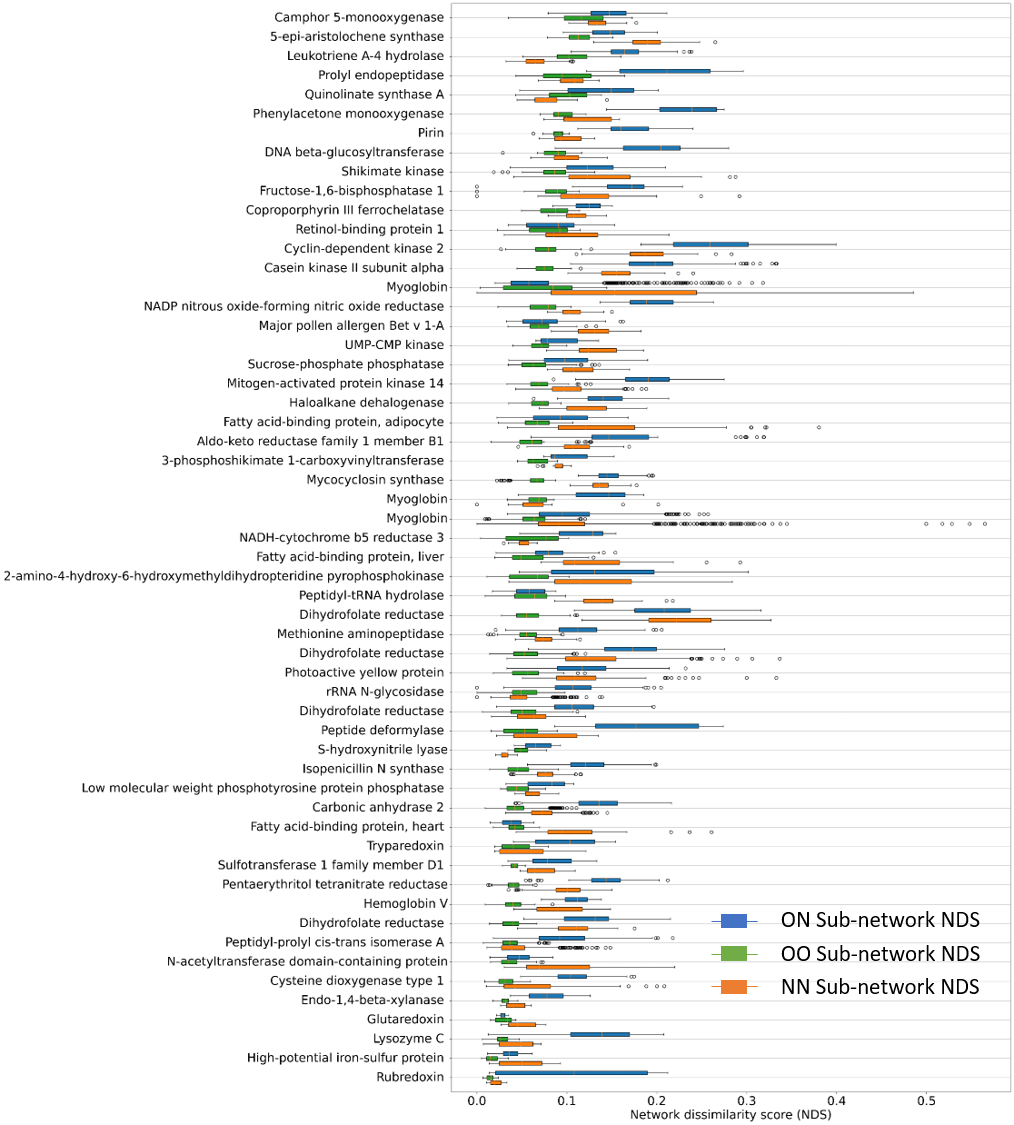


**Appendix (a): Analysis of variability from MD simulation**

MD simulations of Camphor 5-monooxygenase (Cytochrome P450) protein are carried out using GROMACS implementing the CHARMM forcefield to explore non-native conformations.

A pair of crystal structures of the protein, **1YRC** and **5CP4** are used as initial coordinates. The following steps are followed in preparing each of the systems for their simulation.:

- Each of the crystal structures are bound to the co-factor Heme (HEM) and the small molecule Camphor (CAM). The parameter files for the molecules are obtained from CgenFF server.
- The initial structure is placed in a water box with TIP3 water molecules following periodic boundary conditions. The sides of the box are at least 10 Å away from the nearest atom in the protein.
- NA and CL ions are added to neutralise the charges within the box, replacing solvent molecules at random positions. The solvated box containing the small molecule and the cofactor bound to the protein structure are energy minimised before preparing them for simulation.
- Volume equilibration is performed with position restrains using a modified Berendsen thermostat for 100ps, followed by pressure equilibration with position restrains and Parrinello-Rahman barostat for 100ps.
- An initial test run of 50 ns is conducted in triplicates until the structures are stabilised. The simulation with the least structural divergence (average RMSD) is chosen for further simulation.

The chosen simulations are extended for 300 ns each and snapshots of the protein conformers are collected at intervals of 1 ns. The obtained ensemble of **(a) 300 snapshots of simulating 1YRC** and **(b) 300 snapshots of simulating 5CP4** are subject to structural variability analysis by computing their NDS and RMSD between all pair of conformers.

The obtained result of conformational diversity in their simulated snapshots as compared to that obtained from multiple crystal structures is plot in Figure A(a)_1 (A: 1YRC; B: 5CP4). An increased mobility in conformations is observed from the high network dissimilarity (NDS) and high structure difference (RMSD) between simulated snapshots. The temperature effects in the simulations may inherently influence the conformations of the protein, leading to genuine variations in NDS. This is mostly due to higher kinetic energy of the system that allows for it to be more dynamic than multiple conformers captured from crystallisation in their native forms. One other factor that contributes to higher variations can be due to the interaction between atoms of the protein system and their environment.

Figure A(a)_1: Conformational diversity in simulated snapshots as compared to multiple crystal structures. The NDS and RMSD obtained as a result of comparing all pairs of the 300 snapshots in each of the simulation is plot in their respective panels. The NDS and RMSD of all pair of multiple conformers of the Camphor 5-monooxygenase (P00183) protein is also plot in both of the panels to show how much they vary.


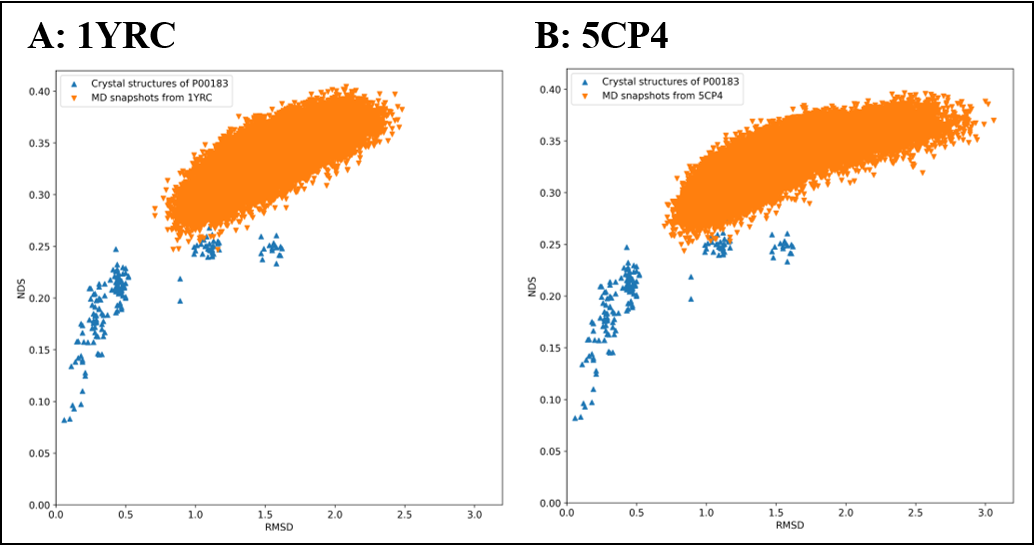


**Appendix (b): Analysis of variability from NMR Structures**

The dataset of crystal structures analysed in our work (Supplementary Table 1) consists of 56 proteins each with more than five multiple crystal structures. We collect all available NMR structures, if any, for all these proteins. In this new dataset of NMR structures, a protein can have more than one NMR structure available. Liver fatty acid binding protein alone has seven NMR structures available which is the highest for a protein in this dataset. 16 proteins in the dataset have 36 NMR structures that are available. The NMR structures report multiple models, each of which is considered as a conformer for this analysis.

All pair of models for each NMR structure are compared to analyse the variability of structure and network across the ensemble. We compute NDS and RMSD for each pairwise comparison and plot the information on a scatter plot as shown in the Figure A(b)_1. The mean RMSD of this scatter is 1.19 Å with a standard deviation of 0.68 Å and the mean NDS is 0.193 with a standard deviation of 0.084 and a Pearson correlation of 0.77 between the NDS and RMSD. These statistics are relatively much higher in the NMR structure analysis than what was observed in the comparison of crystal structures. The correlation between the scores has also improved suggesting that there may be incidental variation in the network proportional to the structural divergence. We do not observe scenarios such as network variation with preserved backbone structure in any of these protein structures.

A table of the mean and standard deviation of NDS and RMSD scores for each protein across crystal and NMR structures that have been analysed is summarised in Table A(b)_1 presented below. The NDS and RMSD scores for each of the 16 proteins that are analysed, show much higher variability in NMR structures as compared to crystal structures (with the exception of MAP kinase protein). For example, the scatter plot of structure and network variation in the liver fatty acid binding protein is shown in Figure A(b)_2.

Figure A(b)_1: All pairs of multiple models from 32 NMR structures are compared to obtain structural (RMSD) and network (NDS) variation information. The NDS and RMSD of proteins in the dataset that have available NMR structures are shown in the scatter plot.


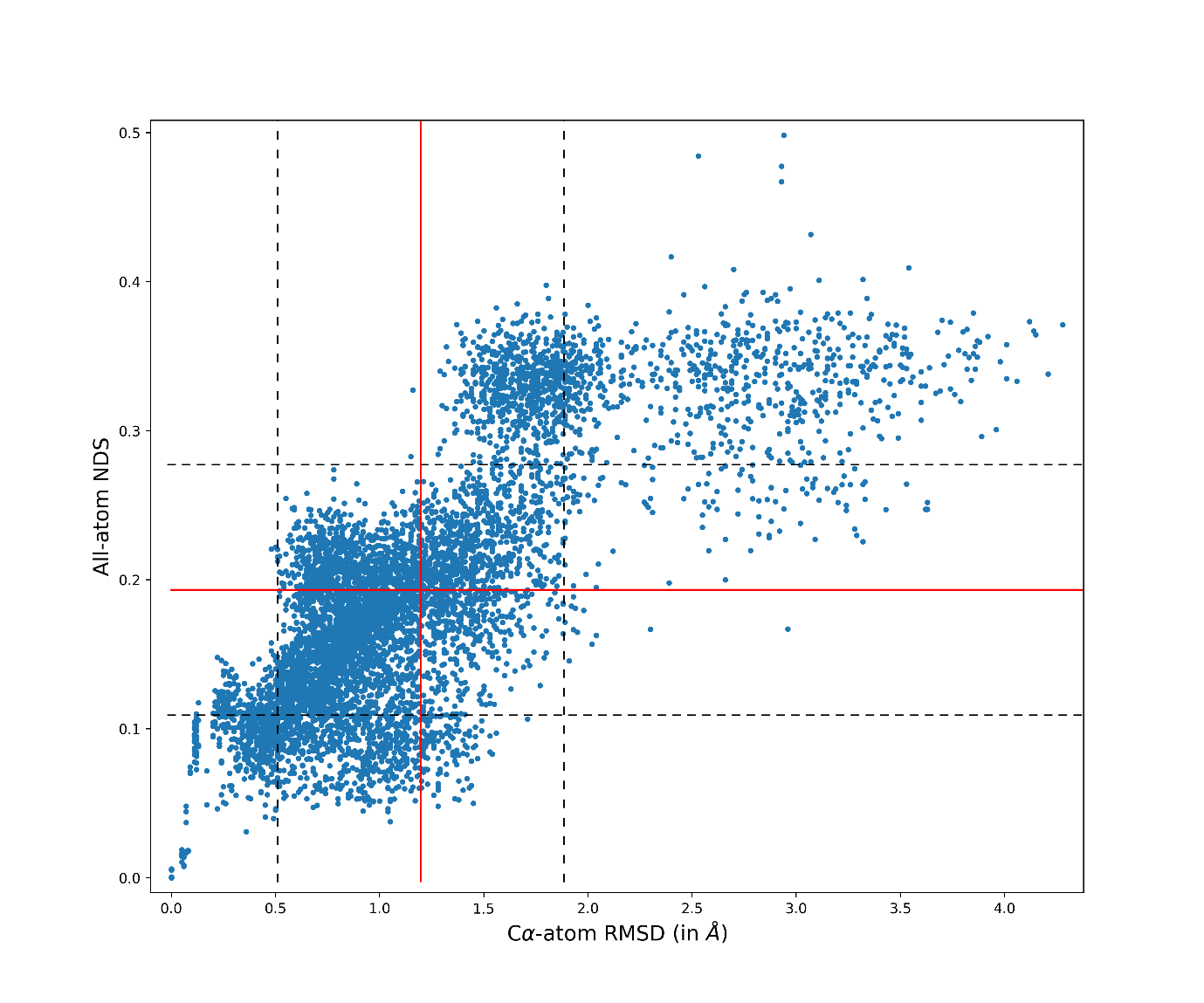


Figure A(b)_2: The conformational diversity of all models of NMR structures of Liver fatty acid binding protein as compared to the conformational diversity from multiple crystal conformers are shown in the scatter plot.


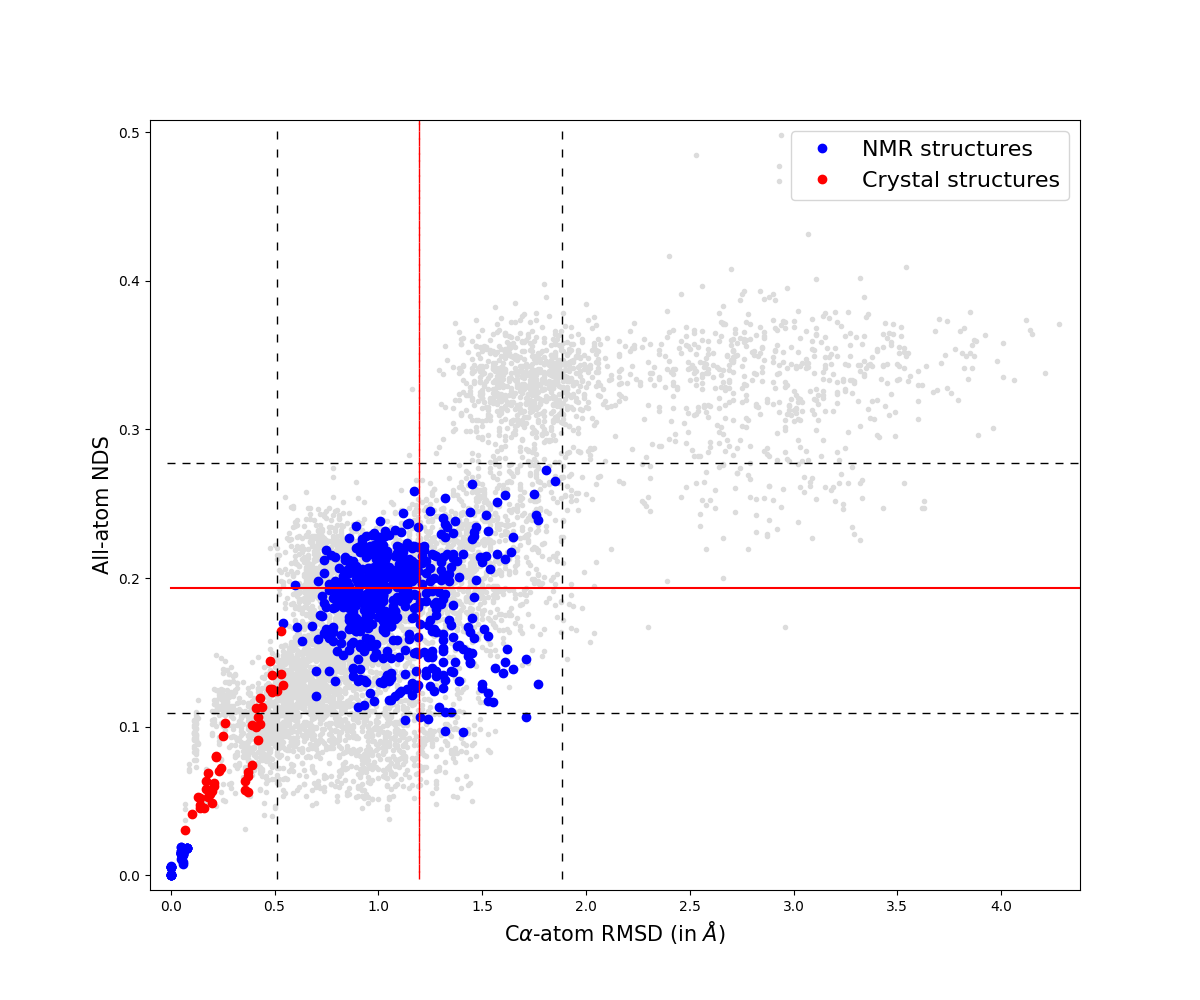


Table A(b)_1: The statistics of RMSD and NDS in all proteins that have available NMR structures are presented in the table. The comparison scores obtained from multiple crystal conformers are provided along with those of models from NMR structures.

| **Proteins** | **Crystal structures** | | **NMR Structures** | |
| --- | --- | --- | --- | --- |
|  | **RMSD** | **NDS** | **RMSD** | **NDS** |
| Heart Fatty acid-binding protein | 0.17 ± 0.06 | 0.056 ± 0.014 | 0.71 ± 0.13 | 0.161 ± 0.02 |
| Lysozyme C | 0.25 ± 0.1 | 0.067 ± 0.025 | 0.74 ± 0.22 | 0.14 ± 0.035 |
| Myoglobin  (Physeter macrocephalus) | 0.39 ± 0.36 | 0.097 ± 0.052 | 1.45 ± 0.3 | 0.206 ± 0.031 |
| 3-phosphoshikimate  1-carboxyvinyltransferase | 0.25 ± 0.14 | 0.136 ± 0.023 | 0.27 ± 0.04 | 0.118 ± 0.012 |
| Dihydrofolate reductase  (Homo sapiens) | 0.49 ± 0.22 | 0.147 ± 0.032 | 0.73 ± 0.11 | 0.212 ± 0.019 |
| Rubredoxin | 0.12 ± 0.06 | 0.046 ± 0.032 | 0.97 ± 0.24 | 0.096 ± 0.025 |
| 2-amino-4-hydroxy-6-hydroxymethyldihydropteridine pyrophosphokinase | 0.86 ± 0.58 | 0.141 ± 0.068 | 1.2 ± 0.37 | 0.218 ± 0.038 |
| Peptide deformylase | 0.38 ± 0.17 | 0.099 ± 0.041 | 0.69 ± 0.55 | 0.124 ± 0.05 |
| Mitogen-activated protein kinase 14 | 1.1 ± 0.39 | 0.174 ± 0.026 | 0.11 ± 0.02 | 0.09 ± 0.016 |
| Peptidyl-prolyl cis-trans isomerase A | 0.28 ± 0.11 | 0.085 ± 0.021 | 1.52 ± 0.4 | 0.295 ± 0.056 |
| Photoactive yellow protein | 0.33 ± 0.17 | 0.102 ± 0.024 | 1.69 ± 0.76 | 0.204 ± 0.069 |
| Liver Fatty acid-binding protein | 0.31 ± 0.14 | 0.084 ± 0.033 | 1 ± 0.35 | 0.172 ± 0.055 |
| Carbonic anhydrase 2 | 0.26 ± 0.12 | 0.116 ± 0.029 | 2.95 ± 0.48 | 0.343 ± 0.02 |
| Major pollen allergen Bet v 1-A | 0.45 ± 0.26 | 0.092 ± 0.028 | 1.21 ± 0.33 | 0.185 ± 0.038 |
